# Childhood obesity and adult cardiovascular disease risk factors: a systematic review with meta-analysis

**DOI:** 10.1186/s12889-017-4691-z

**Published:** 2017-08-29

**Authors:** Amna Umer, George A. Kelley, Lesley E. Cottrell, Peter Giacobbi, Kim E. Innes, Christa L. Lilly

**Affiliations:** 1 Department of Pediatrics, School of Medicine, Robert C. Byrd Health Sciences Center, West Virginia University, Morgantown, West Virginia USA; 2 Department of Biostatistics, School of Public Health, Robert C. Byrd Health Sciences Center, West Virginia University, Morgantown, West Virginia USA; 3 Department of Epidemiology, School of Public Health, Robert C. Byrd Health Sciences Center, West Virginia University, Morgantown, West Virginia USA

**Keywords:** Children, Obesity, Adults, Cardiovascular disease, Systematic review, Meta-analysis

## Abstract

**Background:**

Overweight and obesity is a major public health concern that includes associations with the development of cardiovascular disease (CVD) risk factors during childhood and adolescence as well as premature mortality in adults. Despite the high prevalence of childhood and adolescent obesity as well as adult CVD, individual studies as well as previous systematic reviews examining the relationship between childhood obesity and adult CVD have yielded conflicting results. The purpose of this study was to use the aggregate data meta-analytic approach to address this gap.

**Methods:**

Studies were included if they met the following criteria: (1) longitudinal and cohort studies (including case-cohort), (2) childhood exposure and adult outcomes collected on the same individual over time, (3) childhood obesity, as defined by the original study authors, (4) English-language articles, (5) studies published up to June, 2015, (6) one or more of the following CVD risk factors [systolic blood pressure (SBP), diastolic blood pressure (DBP), total cholesterol (TC), high-density lipoprotein cholesterol (HDL), low-density lipoprotein cholesterol (LDL), non-high-density lipoprotein cholesterol (non-HDL), and triglycerides (TG)], (7) outcome(s) not self-reported, and (8) exposure measurements (child’s adiposity) assessed by health professionals, trained investigators, or self-reported. Studies were retrieved by searching three electronic databases as well as citation tracking. Fisher’s r to *z* score was calculated for each study for each outcome. Pooled effect sizes were calculated using random-effects models while risk of bias was assessed using the STROBE instrument. In order to try and identify sources of heterogeneity, random-effects meta-regression was also performed.

**Results:**

Of the 4840 citations reviewed, a total of 23 studies were included in the systematic review and 21 in the meta-analysis. The findings suggested that childhood obesity is significantly and positively associated with adult SBP (Zr = 0.11; 95% CI: 0.07, 0.14), DBP (Zr = 0.11; 95% CI: 0.07, 0.14), and TG (Zr =0.08; 95% CI: 0.03, 0.13), and significantly and inversely associated with adult HDL (Zr = −0.06; 95% CI: -0.10, −0.02). For those studies that adjusted for adult body mass index (BMI), associations were reversed, suggesting that adult BMI may be a potential mediator. Nine studies had more than 33% of items that placed them at an increased risk for bias.

**Conclusions:**

The results of this study suggest that childhood obesity may be a risk factor for selected adult CVD risk factors. However, a need exists for additional, higher-quality studies that include, but are not limited to, both unadjusted and adjusted measures such as BMI before any definitive conclusions can be reached.

**Systematic review and meta-analysis:**

PROSPERO 2015: CRD42015019763.

**Electronic supplementary material:**

The online version of this article (10.1186/s12889-017-4691-z) contains supplementary material, which is available to authorized users.

## Background

Overweight and obesity during childhood and adolescence is a major public health problem. One of the important health implications of childhood and adolescent obesity includes the development of cardiovascular disease (CVD) risk factors during childhood and adolescence [1–4]. Cardiovascular disease is the leading cause of global mortality [5] accounting for 17.5 million deaths in 2005, and is projected to rise to 23.6 million deaths by 2030 [6]. Several well-established adult CVD risk factors have been identified during childhood. These include, but are not necessarily limited to, high blood pressure (BP), poor lipid profile, impaired glucose tolerance, and metabolic syndrome [7–9]. Importantly, data shows that these risk factors are amplified in the presence of pediatric obesity, referred to by Ford et al. as ‘obesity-associated risk factors for CVD’ [8, 9]. Most notably, a population-based study estimated that 70% of obese children and adolescents between the ages of 5 to 17 have at least one risk factor for CVD [1]. Despite the high prevalence of both childhood and adolescent obesity and adult CVD, studies examining the relationship between childhood obesity and adult CVD have yielded conflicting results [8, 10–13]. This is important given that adult adiposity is an established risk factor for developing adult CVD [14, 15] and there is evidence to suggest that overweight adolescents have a 40%–80% chance of becoming overweight or obese adults [16–18]. Unfortunately, it remains unclear whether childhood obesity is an independent risk factor for adult CVD risk factors or whether childhood obesity persists as adult obesity and indirectly increases the risk of adult CVD [19, 20].

Recent systematic reviews suggest that the relationship between childhood obesity and adult high BP or poor lipid profile is weak, possibly because the results are confounded by adult obesity [13, 21]. In an effort to find previously published systematic reviews and meta-analyses on this topic, a systematic literature search was performed in PubMed on February 2, 2015 (search strategy available in Additional file 1). The search revealed four systematic reviews and one meta-analysis conducted on the relationship between childhood obesity and adult CVD risk factors [13, 21–24] (Additional file 2a). The four systematic reviews published on this topic from 2010 to 2012 provided qualitative evidence but did not provide any quantitative evidence on the association between childhood obesity and adult CVD risk factors (BP and lipid profile). While one meta-analysis was conducted four years ago on this topic, it was limited to a select four cohorts only [24], thereby possibly biasing results. Moreover, this meta-analysis did not calculate the association between childhood obesity and adult total cholesterol (TC) as well as between childhood obesity and adult non-high-density lipoprotein cholesterol (non-HDL) levels. This is important since non-HDL has been shown to be better marker of risk for coronary artery disease and stroke compared to LDL [25, 26]. Of the four systematic reviews, two included hypertension (HT) as one of the main outcomes [22, 23], the third systematic review reported results for resting systolic blood pressure (SBP) and resting diastolic blood pressure (DBP) [21], and the fourth systematic review focused on the lipid profile as the main outcome of interest [13]. With respect to years covered for those studies that included hypertension, Park et al., included studies published between 1980 and 2011 [22], Reilly et al. included studies from January 2002 to mid-June 2010 [23], while Lloyd et al., searched online electronic databases, i.e., PubMed (MEDLINE) and ISI Web of Science from their inception up to July 2008 for the systematic review with hypertension as the outcome [21], and up to July 2010 for their systematic review with serum cholesterol levels as the outcome [13]. In addition, all previous systematic reviews included data where adiposity was measured using BMI for both children and adults [13, 21–24]. However, research has shown that BMI is not an ideal marker for adiposity [27, 28] and including other definitions or classifications of adiposity may help in identifying other potentially eligible studies that have looked at this association. Finally, it appears that the methodological quality of these previous systematic reviews could have been better [13, 21–24]. Using the Assessment of Multiple Systematic Reviews (AMSTAR) Instrument [29], we assessed the methodological quality of the four systematic reviews and one meta-analysis. The overall score for each study ranged from 40% to 80% while scores for each question ranged from 0% to 100% (Additional file 2b). The questions with the three lowest scores included 1) status of publication, 2) including a list of both published and unpublished studies, and 3) assessment for the likelihood of publication bias. These findings provide support for an updated systematic review with meta-analysis on the relationship between childhood obesity and selected adult CVD risk factors, i.e., BP, lipids, and lipoproteins.

Systematic reviews with meta-analysis occupy the highest levels of evidence in the hierarchy of study designs [30]. This structured and standardized approach has been used to make health care decisions and inform policy makers by analyzing prior findings as well as summarizing, synthesizing and critically appraising evidence on a specific topic in the literature [31]. While several systematic reviews [13, 21–23] and one meta-analysis [24] have examined the association between childhood obesity and adult CVD, the investigative team is not aware of any current and thorough systematic review with meta-analysis on this topic. Furthermore, given that childhood obesity as an independent risk factor for CVD in adults is not well-established, the aim of this study was to conduct a systematic review and meta-analysis to critically evaluate the available evidence regarding the association between childhood obesity and select adult CVD risk factors: (1) resting SBP, (2) resting DBP, (3) TC, (4) HDL, (5) LDL, (6) non-HDL, and (7) TG. A secondary aim of the study was to examine whether this association persists after adjusting for adult obesity.

## Methods

This study was conducted and reported according to the Cochrane Collaboration’s recommendations and guidelines for conducting systematic reviews and meta-analyses for observational studies [32, 33] as well as the Preferred Reporting Items for Systematic reviews and Meta-Analyses’ (PRISMA) [34, 35] statement. The study was registered in PROSPERO, an international registry for systematic reviews (Protocol number: PROSPERO 2015:CRD42015019763) [36]. A PRISMA checklist indicating where all items are reported in this study can be found in Additional file 3.

### Study eligibility criteria

The a priori inclusion criteria for this meta-analysis included: (1) longitudinal and cohort studies (including case-cohort), (2) childhood exposure and adult outcomes collected on the same individual over time, (3) main exposure variable of the child’s overweight and obesity status (BMI age-and sex-specific percentiles, percent body fat, fat mass, waist circumference (WC), waist-to-hip ratio, visceral adipose tissue, skin fold thickness, body weight, BMI z-score, BMI or other measures used to assess overweight and obesity in populations) [37], (4) studies available in English-language, (5) studies published up to June, 2015, (6) one or more of the following CVD risk factors as the primary outcome measure: (SBP, DBP, TC, LDL, HDL, non-HDL, and TG), (7) outcome measurements taken by health professionals or trained investigators but not based on self-report data, (8) exposure measurements (child’s adiposity) assessed by health professionals, trained investigators, or self-reported. Exclusion criteria included the following: (1) review articles, (2) cross-sectional study designs, (3) case-control study designs, (4) case reports, (5) comments, (6) letters, (7) animal studies, (8) studies published in non-English language sources, (9) presentations from conference meetings, (10) unpublished studies (abstracts, master theses, dissertations, etc.), and (11) studies in which the outcome(s) were self-reported. Due to limited resources, we did not search the grey literature (unpublished reports, conference abstracts, theses and dissertations, articles in obscure journals, reports, rejected or un-submitted manuscripts) [38] or for studies published in languages other than English. However, we did examine for potential small-study effects (publication bias, etc.) [39, 40]. With respect to meta-analyses that restrict studies to the English language, previous research has shown an overestimation in outcome effects of only 2% [41]. In addition, the percentage of non-English studies traditionally included in meta-analyses is very small [41].

### Data sources

Studies were retrieved using three electronic databases: (1) PubMed (MEDLINE), (2) Web of Science, and (3) Scopus. In addition, cross-referencing from the bibliographies of all retrieved articles (citation tracking) was conducted. An information retrieval specialist (Health Sciences librarian, JS) assisted in the planning of the literature search and in identifying and creating correct Boolean operators and search strings for the different electronic database searches [42]. The PubMed search string used was as follows:

“(obesity OR obese OR overweight OR fat OR adipos* OR “body mass index” OR BMI) AND (child* or adolesc*) AND (“blood pressure” OR hypertension OR cholesterol OR lipid OR lipids OR lipoprotein OR lipoproteins OR cardiovascular) AND (observational OR cohort OR longitudinal) AND adult* AND (human OR humans)”.

Each search was conducted separately and then downloaded as a separate file using Endnote X7 [43]. The first author (AU) conducted all electronic searches and removed all duplicates electronically and then manually. In addition to electronic database searches, cross-referencing from retrieved articles was also performed. A list of all search strategies can be found in Additional file 4.

### Study selection

In order to minimize selection bias, two researchers (AU and CL) independently screened studies for eligibility by reviewing the titles and abstracts of articles based on the pre-defined eligibility criteria. If the inclusion or exclusion criteria could not be decided based on the title and abstract, full-text articles were retrieved and the decision was made accordingly. After independent study selection was performed, the two reviewers met and reviewed every selection for agreement and discrepancies were resolved by consensus. Using Cohen’s kappa statistic, the overall agreement rate prior to correcting discrepancies was 0.75 [44]. If a decision could not be achieved, a content area and meta-analysis expert (GK) resolved any disagreement(s). Multiple publication bias was addressed by including the most recent/relevant study from multiple studies using data from the same cohort.

### Data abstraction

Prior to data abstraction, a detailed codebook that could hold up to 200 items per study was developed by the research team in Microsoft Excel (version 2011) [45]. The codebook included continuous variables, categorical variables, and free text information. The codebook developed was pilot-tested and revised by the investigative team. In order to avoid data abstraction bias, two authors (AU and CL) extracted data from each selected article independently. The researchers then compared every data point for accuracy and consistency until 100% agreement was reached. If agreement could not be reached, a content area and meta-analysis expert (GK) resolved any disagreement(s).

### Risk of bias assessment

Risk of bias was assessed using the Strengthening the Reporting of Observational Studies in Epidemiology (STROBE) instrument [46]. The STROBE instrument consists of a checklist of 22 items that provides guidance on the reporting of observational studies to facilitate critical assessment and interpretation of results [46]. This checklist facilitates assessing the risk of potential bias in the title and abstract, introduction, methods, results, and discussion sections of articles. Each item was classified as “yes” (low risk), “no” (high risk), or “unclear”. An item that was not relevant to an individual study was labeled as “Not Applicable (NA)”. Total scores for each study were adjusted for the NA response. The total number of “yes” (low risk), “no” (high risk), or “unclear” was added and divided by the total number of items for each study and multiplied by 100 in order to report the results in percentages. Based on previous research, no study was excluded based on STROBE scores given the lack of empirical evidence for all currently available instruments, including STROBE, to support such [32, 47–51]. Two researchers (AU and CL) conducted all assessments independent of each other and then examined the results at the study level as well as for each item. The data was then compared for accuracy and consistency. Any disagreements were discussed and resolved until 100% agreement was reached. Using Cohen’s kappa statistic, the overall agreement rate prior to correcting discrepancies was 0.89.

#### Statistical analysis

We conducted an aggregate data meta-analysis for all seven outcomes: (1) SBP, (2) DBP, (3) TC, (4) HDL, (5) LDL, (6) non-HDL and (7) TG. Seven separate Microsoft Excel sheets were generated for all seven outcomes. Any outcome with only one study was excluded from the meta-analysis. Each outcome was further analyzed separately if it was adjusted for adult adiposity.

### Calculation of effect sizes (ES) from each study

The primary outcome for this study was the correlation between childhood adiposity and adult CVD risk factors. The correlation coefficient ‘r’ was transformed using Fishers r to z transformation. The a priori plan was to use risk ratio (RR) as our ES in order to examine the association between childhood obesity and selected adult CVD risk factors. However, because most studies reported a correlation between two continuous variables, a post hoc decision was made to use correlation statistics (Fishers r to z score) instead of RR to serve as the main ES index [52]. All other ESs (odds ratio (OR), mean differences) were converted to correlation statistics using Comprehensive Meta-Analysis (version 3.0) [53].

Standardized beta coefficients from individual studies were used as correlation statistics based on previous research showing that the correlation between a beta coefficient and correlation coefficient is linear, having a correlation of 0.84 if the coefficients reside in the interval ± 0.50 [54]. Studies that presented unstandardized beta-coefficients were first converted to standardized regression coefficients by multiplying the unstandardized coefficient by the ratio of the standard deviations of the independent variable and the dependent variable. Studies where unstandardized regression coefficients could not be converted to standardized regression coefficients and no other ES was provided were excluded from the meta-analysis.

### Pooling of ES’s

Results for the association between childhood obesity and selected CVD risk factors (SBP, DBP, TC, HDL, LDL, non-HDL, and TG) were pooled separately using random-effects, method-of-moments models [55]. The correlation metric was converted to Fisher’s z scale (Fisher’s r-to-z transformation), and all analyses were performed using the transformed values [52]. These results included an overall effect estimate as well as 95% CI [55]. If the 95% CI did not include zero, we considered our results to be statistically significant. Forest plots were used to visually display the estimated ES from each study and their corresponding 95% CI’s. In addition, an overall pooled effect as well as 95% CI’s was generated. Furthermore, 95% prediction intervals (PI’s) for statistically significant results were also calculated.

### Stability and validity of changes in ES’s

Heterogeneity was examined using the Q statistic while inconsistency was assessed using *I* ^*2*^ [56, 57]. Statistical significance for Q was set at an alpha level of ≤0.10 while *I* ^*2*^ was classified as trivial (0%–25%), low (25.1%–50%), moderate (50.1%–75%), or high (75.1%–100%) [57]. Results were also interpreted with respect to the clinical implications of the degree of inconsistency as well as the magnitude and direction across studies, including the strength of evidence for heterogeneity [57].

*Small-study effects* (e.g. publication bias) was examined qualitatively using the funnel plot and quantitatively using Egger’s linear regression test [58]. As recommended by Sterne et al., the test for funnel plot asymmetry was not used when there were fewer than 10 ES [59]. The Egger regression test is a regression of the standardized effect estimates against their precision (inverse standard error) and quantifies funnel plot asymmetry by determining whether the intercept deviates significantly from zero*.* If the intercept was not significantly different from zero, it was assumed that there was no evidence of funnel plot asymmetry [58, 60, 61]*. Influence analysis* with each study deleted from the model once was conducted in order to examine the effects of each study on the overall results [62]. C*umulative meta-analysis*, ranked by the year the study started, was used to examine the accumulation of findings over time [63]. A *sensitivity analysis* was also performed by pooling the ESs from studies that only used childhood BMI as the exposure. This was performed in order to determine if any differences existed in the pooled results that included any definition for childhood exposure, including BMI.

### Meta-regression

Because of missing data for different predictor variables from different studies, simple weighted least squares meta-regression (random-effects, method of moments approach) was used to examine the relationship between each outcome and selected covariates. Meta-regression is analogous to individual study regression except that the outcome variable is the effect estimate, i.e., unit of analysis is the study, rather than individual participant scores [40]. The slope of the regression coefficients along with their 95% confidence intervals (CI) were also calculated. Confidence intervals that did not cross zero were considered statistically significant. Planned covariates to examine a priori included: (1) country in which the study was conducted (USA, other), (2) bias due to loss to follow up, (3) type of analysis, (4) type of definitions used for adiposity, (5) exposure measure (self-report or not), (6) subject characteristics (sex, race/ethnicity), (7) studies that examined the association between childhood obesity and CVD risk factors while controlling or not controlling for adult adiposity, (8) time to follow up, (9) age categories of adults, (10) age categories of children, (11) comorbid conditions for both the child and the adult (diabetes, metabolic syndrome, etc.), (12) lipid lowering medication, (13) hypertensive medication, (14) family history of CVD, (15) smoking status/alcohol or drug use of both the child and the adult, (16) socio-economic status related variables, (17) diet, (18) physical activity, (19) fasting vs. non-fasting lipid profile, (20) child’s pubertal status, (21) perinatal risk factors, and (22) study design. Post-hoc analysis included year of study onset and risk of bias assessment for low-risk studies using the STROBE instrument. When there was insufficient data for potential predictor variables (fewer than 3 results per group), we performed a sensitivity analysis without the predictor to see if it had an effect on our overall findings. For categorical variables, less than three results for any one category were used as the cut-off for analysis. All meta-regression results were considered observational and exploratory, designed to generate hypotheses about potential sources of heterogeneity to be tested in future original studies [40].

## Results

### Study characteristics

**Fig. 1:**
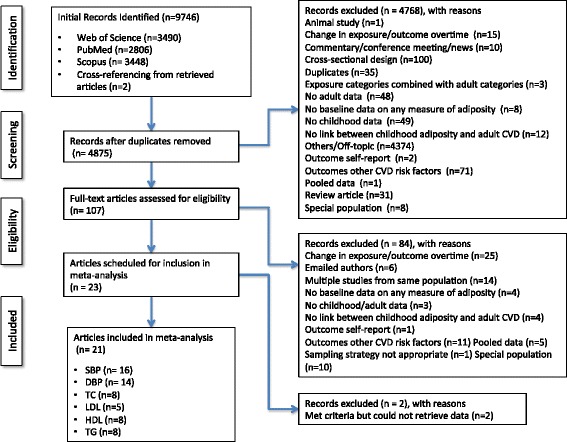
Flow diagram for the selection of studies. Legend: Note: non-HDL was not analyzed because there were less than 3 studies to pool

A general description of the characteristics of each study is shown in Table 1. Of the 4840 citations reviewed, a total of 23 were included in the systematic review [4, 64–85] and 21 in the meta-analysis [4, 64–69, 71–79, 81–85]. A description of the search process, including the reasons for excluded studies, is shown in Fig. 1. A reference list of all excluded studies and reasons for exclusion, by study, are shown in Additional file 5. The year that each study started varied considerably, ranging from 1923 to 1989 while the year that studies were published ranged from 1971 to 2014. Studies were conducted in eleven different countries; six in the U.S. [4, 64, 66, 67, 73, 75], three in United Kingdom (England, Wales, Scotland and Newcastle) [76, 83, 85], three in Finland [65, 68, 81], two in Australia [77, 82], and one in either Sweden [69], India [71], Lithuania [72], Poland [74], the Republic of Seychelles [78], Japan [79], or the Solomon Islands [84]. Most studies used a prospective longitudinal study design except for two studies that used a retrospective study design [65, 73]. None of the studies used a case-cohort study design.

**Table 1:** Study and participant characteristics

Study-cohort name	Study start year	Country	Study design	N Baseline	Age exposure assessed	Age outcome assessed	Exposure	Outcome	Adj. adult BMI	Sex
Abraham et al., 1971 (HMS) [64]	1923	USA	Prospective Cohort	1087	9–12	48	Relative over weight	SBP, DBP, TC	No	Male
Barker et al., 2005 (HBCS) [65]	1934	Finland	Retrospective longitudinal study	2003	2–11 (age used in our study 2)	62	BMI	SBP, TC, TG	Yes (only)	Overall
Berkey et al., 1998 (LSCHD) [66]	1929	USA	Prospective longitudinal study	67	17	30	BMI	SBP	No	Male (this study) Female
Eisenmann et al., 2005 (ACLS) [67]	1970	USA	Longitudinal, prospective epidemiological study	48	16	27	BMI, WC, %BF	SBP, DBP, TC, HDL, TG	No	Overall
Freedman et al., 2001 (BHS) [4]	1973	USA	Cross panel design later longitudinal component	2617	10	27	BMI, Triceps ST	SBP, DBP, TC, LDL, HDL, TG	Yes	Overall
Graversen et al., 2014 (NFBC 1966) [68]	1966	Finland	Population based cohort	4111	2–5 (age used in our study 5)	31	BMI	SBP, DBP, HDL, TG	No	Overall
Gustafsson et al., 2011 (NSC) [69]	1981	Sweden	Prospective cohort study	1083; F = 506 M = 577	16	21 (this study), 30, 43	BMI	SBP, DBP	No	Male Female
Kanade et al., 2011 (CBCI) [71]	1979	India	Community base prospective cohort study	387	3, 15	24	BMI	SBP, DBP, TG	No	Male
Klumbiene et al., 2000 (JHL) [72]	1977	Lithuania	Longitudinal cohort	505 M = 217 F = 288	12–13	32–33	BMI, Triceps ST, Sub-scapular ST	SBP, DBP	No	Male Female
Kneisley et al., 1990 (BPS-TM) [73]	1959	USA	Retrospective Cohort Study	576 M = 271 F = 305	7	32	Sub-scapular ST	SBP	No	Male Female
Koziel et al., 2011 (WGS) [74]	1961	Poland	Longitudinal Study	M = 124 F = 139	8, 9, 10, 11, 12, 13, 14, 15, 16, 17, 18	50	BMI	SBP	Yes (only)	Male Female
Holland et al., 1993 (NSHD) [70]^a^	1946	England, Wales, Scotland	Prospective longitudinal study	3332 at birth	4–7, 11–14	36	BMI	SBP, DBP	No	Male Female
Lauer et al., 1993 (MS) [75]^b^	1971	USA	Longitudinal Cohort	M = 677 F = 748 M = 492 F = 528	7–8, 9–10, 11–12, 13–14, 15–16, 17–18; 13–14, 15–16, 17–18	20–25, 26–30	BMI	SBP, DBP	No	Male Female
Li et al., 2007 (BBC- 1958) [76]	1958	England, Wales and Scotland	Longitudinal, Birth Cohort	9297	7, 11, 16	33, 45 (this study)	BMI	SBP, DBP	Yes	Overall
Liddle et al., 2012 (MUSP) [77]	1981	Australia	Longitudinal Birth Cohort study	1755	5	21	BMI, Triceps ST	SBP, DBP	No	Overall
Lyngdoh et al., 2013 (SCDC) [78]	1989	The Republic of Seychelles	Longitudinal cohort	390 M = 175 F = 215	12–15	19–20	BMI	SBP, DBP, LDL, HDL, TG	Yes	Overall Male Female
Miura et al., 2001 (YAJS) [79]	1965	Japan	20-year FU data using record linkage of a Birth cohort	M = 2198 F = 2428	3	20	BMI	SBP, DBP, TC	No	Male Female
^^^Pereira et al., 2013 (BBC- 1958) [80]	1958	England, Wales and Scotland	Large Population based Birth Cohort	M = 3927 F = 3897	7, 11, 16	23, 33, 42, 45	BMI	TC, LDL, HDL, TG, non-HDL	No	Male Female
Porkka et al., 1994 (YFS) [81]	1980	Finland	Longitudinal Cohort	3596	3–9, 12–18 (this study)	24–30	BMI, Sub-scapular ST	TC, LDL, HDL, TG	No	Male Female
Schmidt et al., 2011 (ASHFS-CDAHS) [82]	1985	Australia	Prospective cohort study	2188	7–15	26–32	BMI, WC, WHR, weight to height ratio, sum ST	SBP, DBP, HDL, TG	No	Overall
Skidmore et al., 2007 (NSHD) [83]	1946	England, Wales and Scotland	Prospective longitudinal birth cohort study	5362 births (2311 for the analysis)	2, 4, 7, 11, 15	53	BMI	TC, LDL, HDL	No	Overall
Weitz et al., 2014 (LS-Mid-TC) [84]	1966	Six Solomon Island	Longitudinal	540 M = 169 F = 219	0–5, 6–11, 12–19 (6–11, 12–19 for this study)	25	BMI, Sub-scapular ST	TC, TG	No	Male Female
Wright et al., 2001 (NTFCS) [85]	1947	UK	Prospective birth cohort	1142 at birth, 2/3rd followed till age 15	9, 13	50		SBP, DBP, TC, LDL, HDL, TG	Yes	Male Female

*SBP* systolic blood pressure; *DBP* diastolic blood pressure; *TC* total cholesterol; *HDL* high-density lipoprotein cholesterol; *LDL* low-density lipoprotein cholesterol; *non*-*HDL* non-high-density lipoprotein cholesterol; *TG* triglycerides; *BMI* body mass index; *WC* waist circumference; *ST* skinfold thickness; *BF* body fat; *WHR* waist to hip ratio; *ACLS* Aerobics Center Longitudinal Study; *ASHFS*-*CDAHS* Australian Schools Health and Fitness Survey-Childhood Determinants of Adult Health Study; *BHS* Bogalusa Heart Study; *BPS*-*TM* Blood Pressure Study in Tecumseh Michigan; *BBC*-*1958* British Birth Cohort 1958; *CBCI*, Community Based Cohort study India; *HMS* Hagerstown Morbidity Study; *HBCS* Helsinki Birth Cohort Study; *JHL* Study of Juvenile Hypertension in Lithuania; *LSCHD* Longitudinal Study of Child Health and Development; *LS*, *Mid*-*TC* Longitudinal Study of the Mid-20th Century; *MS* Muscatine Study; *MUSP* Mater-University of Queensland Study of Pregnancy; *NSC* Northern Swedish Cohort; *NFBC 1966*, Northern Finland Birth Cohort 1966 Study; *NSHD*, Medical Research Council National Survey of Health and Development; *NTFCS* Thousand Families Cohort Study; *SCDC* Seychelles Child Development Study; *YFS* Cardiovascular Risk in Young Finns; *WGS* Wroclaw Growth Study; *YAJS* Young Adult Japanese Study

^a^Holland et al., 1993 and Pereira et al., 2013 not included in the meta-analysis

^b^Lauer et al., 1993 (MS) Adults who had their blood pressure measured during their school years were recalled for re-exam at ages 23 or 28. Children with data from 2 adult intervals: 20–25 and 26–30 was used in this study

The length of follow-up for the studies ranged from 4.5 to 60 years. As most of the studies were prospective longitudinal studies, the number of subjects at baseline was often greater than the number of participants at follow-up due to loss at follow-up. Seven studies included information on loss to follow-up [4, 68, 69, 71, 72, 77, 82]. Reasons for ‘lost to follow-up’ included the following: (1) refused to participate, (2) inability to locate, (3) did not respond to contact, (4) participants out of country or town at time of follow-up, (5) death, (6) difficult to contact married girls in India who left their native village, (7) social disadvantage (less well educated and having lower family income). Two studies specified that participants who were lost to follow-up did not have significantly different childhood BMI’s when compared to those who were available at follow-up in adulthood [4, 85].

With respect to exposure variable measurements, none were self-reported. Most studies used BMI as a measure of adiposity in addition to other measures used [4, 65–69, 71, 72, 74–79, 81–85]. However, two studies used relative overweight [64] and sub-scapular skinfold thickness measures only [73]. Most studies did not use a cut-off point to define childhood obesity, but rather, used childhood BMI as a continuous variable.

Sixteen studies examined the association between childhood obesity and adult SBP [4, 64, 66–69, 71–73, 75–79, 82, 85], 14 examined the association between childhood obesity and adult DBP [4, 64, 67–69, 71, 72, 75–79, 82, 85], 8 examined the association between childhood obesity and adult TC [4, 64, 67, 79, 81, 83–85], 5 examined the association between childhood obesity and adult LDL [4, 78, 81, 83, 85], 8 examined the association between childhood obesity and adult HDL [4, 67, 68, 78, 81–83, 85], and 9 examined the association between childhood obesity and adult TG [4, 67, 68, 71, 78, 81, 82, 84, 85]. However, data from one study that assessed TG could not be used for meta-analysis because the study’s main outcome was blood pressure and there was not enough information provided for TGs to calculate an ES [71]. One study by Pereira et al., included data for the association between childhood adiposity and adult non-HDL cholesterol [80]. However, this study was excluded from the meta-analysis due to a lack of data to calculate the standardized regression coefficients based on the information provided. Another study by Holland and colleagues was excluded from the meta-analysis for the same reason [70]. Of the 21 studies, only six (28.6%) included data on the association between childhood obesity and adult CVD risk factors while adjusting for adult BMI [4, 65, 74, 76, 78, 85]. Two of these six studies reported adjusted associations only [65, 74].

### Participant characteristics

As previously stated, a description of the participant characteristics is shown in Table 1. The majority of studies included information on both males and females [66, 69, 72–75, 78, 79, 81, 84, 85]; two were limited to men only [64, 71] while 9 included combined data for both men and women [4, 65, 67, 68, 76–78, 82, 83]. No study was limited to women. One study included combined results as well as results according to sex [78]. However, for the current meta-analysis, we used the results reported according to sex [78]. Moreover, while one study had data on both males and females, data for only males was used for the current meta-analysis because the regression model for females included change of BMI over time i.e., from childhood to adulthood [66].

The participants’ ages at baseline when the exposure was measured ranged from 2 to 18 years. The age at follow-up when the outcome was measured also varied substantially between studies, ranging from 19 to 62 years. Some studies used one childhood age or an average of a range of childhood ages as their exposure, while other studies categorized children based on different age groups. Furthermore, some studies used longitudinal data for the same children over time.

Most studies provided some level of information on comorbid conditions as adults. These included hypertension [64], arteriosclerotic heart disease [64], CV renal disease [64], coronary heart disease [65], diabetes, insulin or glucose levels [4, 68, 78], metabolic syndrome [69], medication for heart diseases [76], medication for hypertension [68, 76] [74] and uric acid level [78]. One study excluded participants who were on hypertensive (HT) medication [73], whereas one study found no difference in any of the analyses after performing sensitivity analysis and excluding those subjects who were taking cholesterol-lowering drugs [83]. For women, additional information on the use of oral contraceptives, menstruation, menopausal status, and use of hormonal replacement therapy was provided by five studies [74–76, 81, 83]. Family history of CVD or CVD risk factors was available in 2 studies [73, 75]. Information on smoking, alcohol and drug use was provided by six studies [69, 71, 75, 76, 81, 82]. Perinatal risk factors such as birth weight and/or gestational age were presented in approximately one third of the studies [66, 68, 76, 77, 79, 83, 85]. Information on diet and physical activity was provided by four studies [69, 76, 81, 82], while information on the child’s pubertal status was provided by one study [72]. It is important to note that these variables were not necessarily adjusted for in the analyses performed by the individual studies.

### Risk of bias assessment

**Fig. 2:**
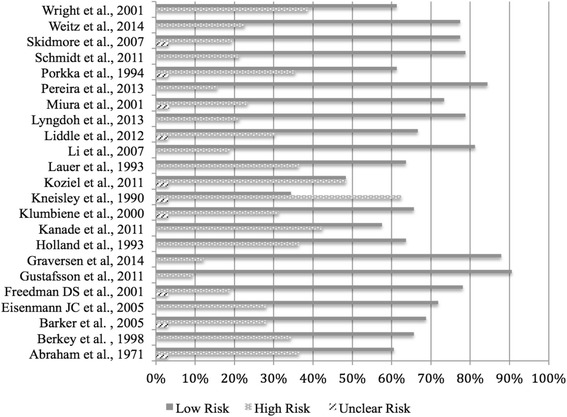
Risk of bias assessment using STROBE instrument for each study. Legend: STROBE-Strengthening the Reporting of Observational Studies in Epidemiology (STROBE) instrument is a checklist of 22 items related to the title, abstract, introduction, methods, results and discussion sections of the articles. Each study was assessed for the 22 items. The total number of “yes” (low risk), “no” (high risk), or “unclear” items were added and divided by the total number of items for each study and multiplied by 100 in order to report the results in percentages. Scores were adjusted for NA responses. The STROBE checklist is available at: http://strobe-statement.org/index.php?id=available-checklists

Overall study-level risk of bias results are shown in Fig. 2 while results for each item from each study are shown in Additional file 6. More than 50% of the studies did not provide an adequate description of participant characteristics while almost 70% did not describe any efforts to address potential sources of bias, explain how loss to follow-up was addressed, or provide reasons for non-participation at each stage. More than 80% did not explain how missing data was addressed. At the study level, nine studies had more than one third of items that were at an increased risk of bias [64, 66, 70, 71, 73–75, 81, 85].

### Primary outcomes

**Fig. 3:**
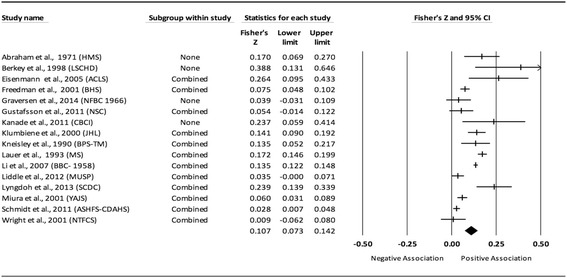
Forest plot for the association between childhood obesity and adult SBP. Legend: The common metric for the effect size for each study is the Fisher’s r to z transformation of the correlation statistics. The vertical lines in the middle of each straight line represent the mean of Fisher’s Z while the left and right extremes of the vertical lines represent the corresponding 95% CI. The middle of the black diamond represents the overall mean for Fishers Z while the left and right extremes of the diamond represent the corresponding 95% CI. Combined measures represent those studies in which males and females were combined, different age cohorts from each study were combined, multiple readings from the same cohort were combined, or one study using more than one exposure definition was combined

**Fig. 4:**
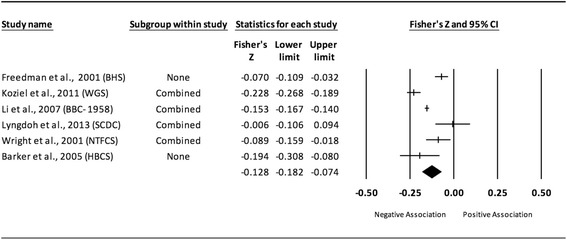
Forest plot for the association between childhood obesity and adult SBP (adjusted for adult BMI). Legend: The common metric for the effect size for each study is the Fisher’s r to z transformation of the correlation statistics. The common metric for the effect size for each study is the Fisher’s r to z transformation of the correlation statistics. The vertical lines in the middle of each straight line represent the mean of Fisher’s Z while the left and right extremes of the vertical lines represent the corresponding 95% CI. The middle of the black diamond represents the overall mean for Fishers Z while the left and right extremes of the diamond represent the corresponding 95% CI. Combined measures represent those studies in which males and females were combined, different age cohorts from each study were combined, multiple readings from the same cohort were combined, or one study using more than one exposure definition

**Fig. 5:**
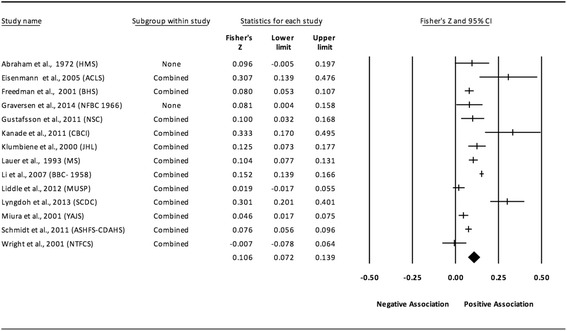
Forest plot for the association between childhood obesity and adult DBP. Legend: The common metric for the effect size for each study is the Fisher’s r to z transformation of the correlation statistics. The vertical lines in the middle of each straight line represent the mean of Fisher’s Z while the left and right extremes of the vertical lines represent the corresponding 95% CI. The middle of the black diamond represents the overall mean for Fishers Z while the left and right extremes of the diamond represent the corresponding 95% CI. Combined measures represent those studies in which males and females were combined, different age cohorts from each study were combined, multiple readings from the same cohort were combined, or one study using more than one exposure definition

**Fig. 6:**
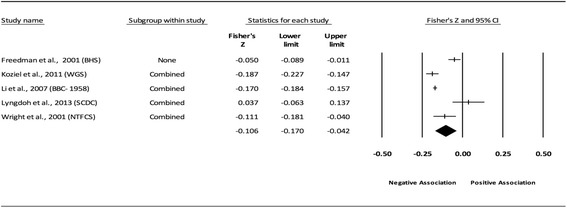
Forest plot for the association between childhood obesity and adult DBP (adjusted for adult BMI). Legend: The common metric for the effect size for each study is the Fisher’s r to z transformation of the correlation statistics. The vertical lines in the middle of each straight line represent the mean of Fisher’s Z while the left and right extremes of the vertical lines represent the corresponding 95% CI. The middle of the black diamond represents the overall mean for Fishers Z while the left and right extremes of the diamond represent the corresponding 95% CI. Combined measures represent those studies in which males and females were combined, different age cohorts from each study were combined, multiple readings from the same cohort were combined, or one study using more than one exposure definition

**Fig. 7:**
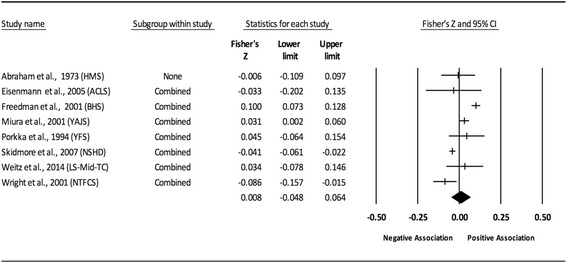
Forest plot for the association between childhood obesity and adult TC. Legend: The common metric for the effect size for each study is the Fisher’s r to z transformation of the correlation statistics. The vertical lines in the middle of each straight line represent the mean of Fisher’s Z while the left and right extremes of the vertical lines represent the corresponding 95% CI. The middle of the black diamond represents the overall mean for Fishers Z while the left and right extremes of the diamond represent the corresponding 95% CI. Combined measures represent those studies in which males and females were combined, different age cohorts from each study were combined, multiple readings from the same cohort were combined, or one study using more than one exposure definition

**Fig. 8:**
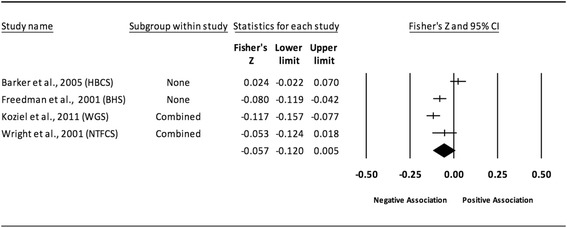
Forest plot for the association between childhood obesity and adult TC (adjusted for adult BMI). Legend: The common metric for the effect size for each study is the Fisher’s r to z transformation of the correlation statistics. The vertical lines in the middle of each straight line represent the mean of Fisher’s Z while the left and right extremes of the vertical lines represent the corresponding 95% CI. The middle of the black diamond represents the overall mean for Fishers Z while the left and right extremes of the diamond represent the corresponding 95% CI. Combined measures represent those studies in which males and females were combined, different age cohorts from each study were combined, multiple readings from the same cohort were combined, or one study using more than one exposure definition

**Fig. 9:**
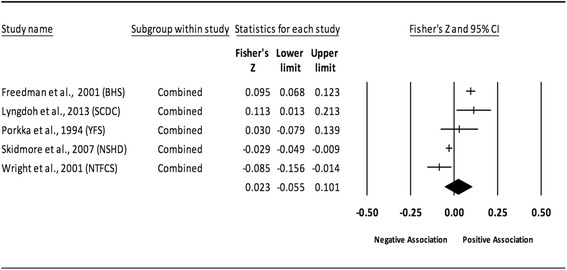
Forest plot for the association between childhood obesity and adult LDL. Legend: The common metric for the effect size for each study is the Fisher’s r to z transformation of the correlation statistics. The vertical lines in the middle of each straight line represent the mean of Fisher’s Z while the left and right extremes of the vertical lines represent the corresponding 95% CI. The middle of the black diamond represents the overall mean for Fishers Z while the left and right extremes of the diamond represent the corresponding 95% CI. Combined measures represent those studies in which males and females were combined, different age cohorts from each study were combined, multiple readings from the same cohort were combined, or one study using more than one exposure definition

**Fig. 10:**
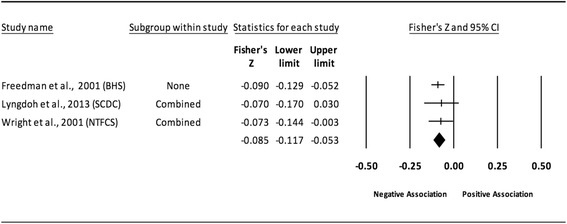
Forest plot for the association between childhood obesity and adult LDL (adjusted for adult BMI). Legend: The common metric for the effect size for each study is the Fisher’s r to z transformation of the correlation statistics. The vertical lines in the middle of each straight line represent the mean of Fisher’s Z while the left and right extremes of the vertical lines represent the corresponding 95% CI. The middle of the black diamond represents the overall mean for Fishers Z while the left and right extremes of the diamond represent the corresponding 95% CI. Combined measures represent those studies in which males and females were combined, different age cohorts from each study were combined, multiple readings from the same cohort were combined, or one study using more than one exposure definition

**Fig. 11:**
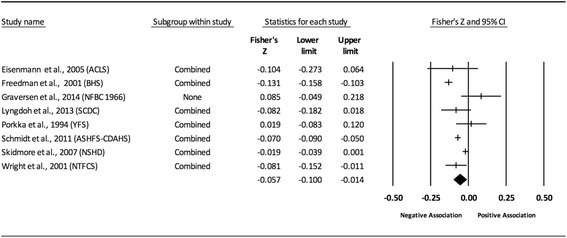
Forest plot for the association between childhood obesity and adult HDL. Legend: The common metric for the effect size for each study is the Fisher’s r to z transformation of the correlation statistics. The vertical lines in the middle of each straight line represent the mean of Fisher’s Z while the left and right extremes of the vertical lines represent the corresponding 95% CI. The middle of the black diamond represents the overall mean for Fishers Z while the left and right extremes of the diamond represent the corresponding 95% CI. Combined measures represent those studies in which males and females were combined, different age cohorts from each study were combined, multiple readings from the same cohort were combined, or one study using more than one exposure definition

**Fig. 12:**
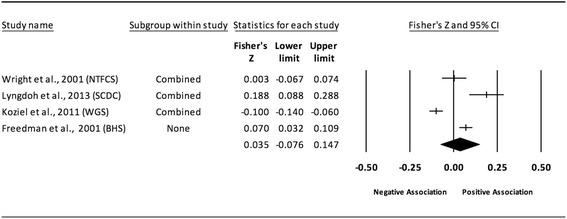
Forest plot for the association between childhood obesity and adult HDL (adjusted for adult BMI). Legend: The common metric for the effect size for each study is the Fisher’s r to z transformation of the correlation statistics. The vertical lines in the middle of each straight line represent the mean of Fisher’s Z while the left and right extremes of the vertical lines represent the corresponding 95% CI. The middle of the black diamond represents the overall mean for Fishers Z while the left and right extremes of the diamond represent the corresponding 95% CI. Combined measures represent those studies in which males and females were combined, different age cohorts from each study were combined, multiple readings from the same cohort were combined, or one study using more than one exposure definition

**Fig. 13:**
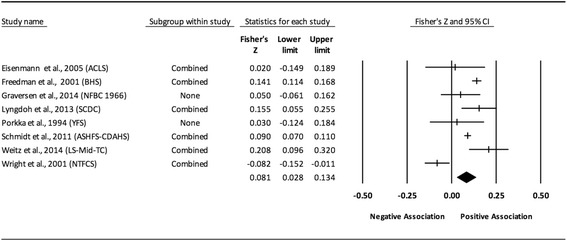
Forest plot for the association between childhood obesity and adult TG. Legend: The common metric for the effect size for each study is the Fisher’s r to z transformation of the correlation statistics. The vertical lines in the middle of each straight line represent the mean of Fisher’s Z while the left and right extremes of the vertical lines represent the corresponding 95% CI. The middle of the black diamond represents the overall mean for Fishers Z while the left and right extremes of the diamond represent the corresponding 95% CI. Combined measures represent those studies in which males and females were combined, different age cohorts from each study were combined, multiple readings from the same cohort were combined, or one study using more than one exposure definition

**Fig. 14:**
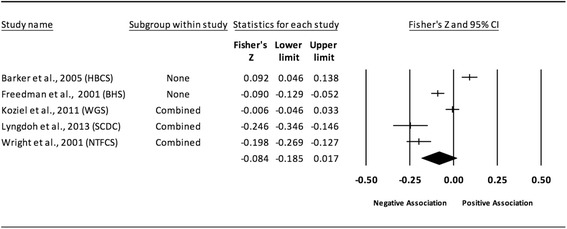
Forest plot for the association between childhood obesity and adult TG (adjusted for adult BMI). Legend: The common metric for the effect size for each study is the Fisher’s r to z transformation of the correlation statistics. The vertical lines in the middle of each straight line represent the mean of Fisher’s Z while the left and right extremes of the vertical lines represent the corresponding 95% CI. The middle of the black diamond represents the overall mean for Fishers Z while the left and right extremes of the diamond represent the corresponding 95% CI. Combined measures represent those studies in which males and females were combined, different age cohorts from each study were combined, multiple readings from the same cohort were combined, or one study using more than one exposure definition

The results of the random-effects meta-analysis for the association between childhood obesity and adult CVD risk factors are presented in Tables 2 and 3 while forest plots displaying the effect estimates along with the 95% CI for each outcome are shown in Figs. 3, 4, 5, 6, 7, 8, 9, 10, 11, 12, 13 and 14. The forest plots for influence analyses, cumulative meta-analysis, and funnel plots are shown in Additional file 7. Exploratory random-effects meta-regression analyses for the association between childhood obesity and adult CVD risk factors and selected covariates (categorical and continuous) in which adequate data were available are shown in Additional file 8.

**Table 2:** Changes in primary outcomes using any definition for adiposity

**Variable**	**Studies (#)**	**Participants (#)**	**Zr (95% CI)**	**Q (p)**	***I*** ^***2***^ ***(*** **%)**	**95% PI**
SBP	16	27,487	0.11 (0.07, 0.14)*	162.44 (<0.001)**	90.77	−0.03, 0.23
SBP (adjusted)	6	15,156	−0.13 (−0.18, −0.07)*	43.05 (<0.001)**	88.39	−0.31, 0.02
DBP	14	27,153	0.11 (0.07, 0.14)*	135.95 (<0.001)**	90.44	−0.01, 0.23
DBP (adjusted)	5	13,356	−0.11 (−0.17, −0.04)*	51.75 (<0.001)**	92.27	−0.37, 0.06
TC	8	10,420	0.01 (−0.05, 0.06)	79.69 (<0.001)**	91.22	−0.16, 0.18
TC (adjusted)	4	7272	−0.06 (−0.12, 0.01)	21.72 (<0.001)**	86.19	−0.32, 0.19
LDL	5	5462	0.02 (−0.06, 0.10)	63.07 (<0.001)**	93.66	−0.25, 0.27
LDL (adjusted)	3	3365	−0.08 (−0.12, −0.05)*	0.31 (0.855)	0	−0.29, 0.12
HDL	8	7915	−0.06 (−0.10, −0.02)*	51.65 (<0.001)**	86.44	−0.18, 0.06
HDL (adjusted)	4	5854	0.04 (−0.08, 0.15)	50.40 (<0.001)**	94.05	−0.47, 0.47
TG	8	5919	0.08 (0.03, 0.13)*	42.0 (<0.001)**	83.33	−0.05, 0.25
TG (adjusted)	5	5854	−0.08 (−0.19, 0.02)	76.20 (<0.001)**	94.75	−0.40, 0.31

*SBP* systolic blood pressure; *DBP* diastolic blood pressure; *TC* total cholesterol; *LDL* low-density lipoprotein cholesterol; *HDL* high-density lipoprotein cholesterol; *TG* triglycerides; Adjusted, adjusted for adult body mass index (BMI); Zr, Fisher’s z scale (Correlation coefficient ‘r’ transformed using Fisher’s z transformation to achieve normal sampling distribution; Q, Cochran’s Q statistic; *I* ^*2*^ *,* I-squared, calculated as 100% × (Q - df)/Q, where df is degrees of freedom; *I* ^*2*^ classified as trivial (0%–25%), low (25.1%–50%), moderate (50.1%–75%), or high (75.1%–100%) [57]; PI, prediction intervals; **boldfaced** items are statistically significant

*statistically significant (non-overlapping 95% CI)

**statistically significant at *p* < 0.10

**Table 3:** Changes in primary outcomes limited to BMI for childhood exposure

**Variable**	**Studies (#)**	**Zr (95% CI)**	**Q (P)**	***I*** ^***2***^ **(%)**	**95% PI**
SBP	14	0.10 (0.06, 0.13)*	97.56 (<0.001)**	86.68	0, 0.23
DBP	13	0.11 (0.07, 0.14)*	100.19 (<0.001)**	88.02	0, 0.24
TC	7	0.01 (−0.05, 0.07)	54.28 (<0.001)**	88.95	−0.17, 0.16
LDL	5	0.02 (−0.06, 0.10)	63.07 (<0.001)**	93.66	−0.28, 0.28
HDL	8	−0.06 (−0.11, −0.01)*	39.61 (<0.001)**	82.33	−0.20, 0.09
TG	7	0.10 (0.02, 0.17)*	42.0 (<0.001)**	83.33	−0.13, 0.34

*SBP* systolic blood pressure; *DBP* diastolic blood pressure; *TC* total cholesterol; *LDL* low-density lipoprotein cholesterol; *HDL* high-density lipoprotein cholesterol; *TG* triglycerides; All studies adjusted for adult body mass index (BMI) and limited to BMI as the exposure; Zr, Fisher’s z scale (Correlation coefficient ‘r’ is transformed using Fisher’s z transformation to achieve normal sampling distribution; Q, Cochran’s Q statistic; *I* ^*2*^ *,* I-squared, calculated as 100% × (Q - df)/Q, where df is degrees of freedom; *I* ^*2*^ classified as trivial (0%–25%), low (25.1%–50%), moderate (50.1%–75%), or high (75.1%–100%) [57]; PI, prediction intervals; **boldfaced** items are statistically significant

*statistically significant (non-overlapping 95% CI)

**statistically significant at *p* < 0.10

#### Systolic blood pressure (unadjusted for adult adiposity)

Overall, there was a statistically significant and positive association between childhood adiposity and adult SBP (Table 2 and Fig. 3). Heterogeneity was also statistically significant and large. However, 95% PIs were non-significant. Funnel plot results for small-study effects showed a lack of asymmetry and were reinforced by a lack of statistical significance based on Egger et al.’s regression intercept test (*p* = 0.42). With each study deleted from the model once, results remained statistically significant across all deletions, ranging from 0.10 to 0.11. Cumulative meta-analysis, ranked by the year the study started, demonstrated that results have been statistically significant since examining the birth cohort of 1958. Random-effects meta-regression revealed statistically significant evidence for an association between pooled ES (Fisher’s r to z score for the association between childhood obesity and adult SBP) and baseline age (β = 0.01, *p* = 0.01; *I* ^*2*^ reduced from 91% to 88%) only.

#### Systolic blood pressure (adjusted for adult adiposity)

When examining studies that adjusted for adult BMI, a statistically significant and negative association was observed between childhood adiposity and adult SBP (Table 2 and Fig. 4). Heterogeneity was also statistically significant and considered large. However, 95% PIs were non-significant. Because there were less than 10 studies, small-study effects were not assessed. With each study deleted from the model once, results remained statistically significant across all deletions. The associations ranged from approximately −0.10 to −0.15. Cumulative meta-analysis, ranked by the year the studies started, demonstrated that results have been statistically significant since the first study was conducted in 1934. Random-effects meta-regression revealed statistically significant evidence for an association between pooled ES (Fisher’s r to z score for the association between childhood obesity and adult SBP for studies that adjusted for adult BMI) and follow-up age (β = −0.005, *p* = 0.002; *I* ^*2*^ reduced from 89% to 69%) as well as length of follow-up (follow-up age – baseline age) (β = −0.004, *p* = 0.008; *I* ^*2*^ reduced from 89% to 76%).

#### Diastolic blood pressure (unadjusted for adult adiposity)

Overall, there was a statistically significant and positive association between childhood adiposity and adult DBP (Table 2 and Fig. 5). Heterogeneity was also statistically significant and large. However, 95% PIs were non-significant. Funnel plot results for small-study effects showed a lack of asymmetry and were reinforced by a lack of statistical significance based on Egger et al.’s regression intercept test (*p* = 0.37). With each study deleted from the model once, results remained statistically significant across all deletions. The associations ranged from 0.09 to 0.11. Cumulative meta-analysis, ranked by the year the study started, demonstrated that results have been significant since examining the birth cohort of 1966. Random-effects meta-regression revealed statistically significant evidence for an association between pooled ES (Fisher’s r to z score) for the association between childhood obesity and adult DBP and baseline age (β = 0.01, *p* = 0.01; *I* ^*2*^ reduced from 90% to 87%).

#### Diastolic blood pressure (adjusted for adult adiposity)

When examining studies that adjusted for adult BMI, a statistically significant and negative association was observed between childhood adiposity and adult DBP (Table 2 and Fig. 6). Heterogeneity was also statistically significant and considered large. However, 95% PIs were non-significant. Because there were less than 10 studies, small-study effects were not assessed. The results of the influence analysis showed that when two studies (Koziel et al., 2011 [74] and Li et al., 2007 [76]) were deleted separately from the model, results were slightly non-significant. The results remained statistically significant when deleting the rest of the studies individually. The associations ranged from approximately −0.08 to −0.13. Cumulative meta-analysis, ranked by the year the studies started, demonstrated that results have been statistically significant since the first study was conducted in 1947. Random-effects meta-regression revealed statistically significant evidence for an association between pooled ES (Fisher’s r to z score for the association between childhood obesity and adult DBP for studies that adjusted for adult BMI) and follow-up age (β = −0.006, *p* < 0.0001; *I* ^*2*^ reduced 92% to 58%), length of follow-up (β = −0.006, *p* < 0.0001; *I* ^*2*^ reduced from 92% to 56%) as well as year of study onset (β = 0.0047, *p* = 0.0122; *I* ^*2*^ reduced from 92% to 82%).

#### Total cholesterol (unadjusted for adult adiposity)

Overall, there was a positive association between childhood adiposity and adult TC (Table 2 and Fig. 7). However, the association was not statistically significant. The 95% PIs were also non-significant. Heterogeneity was statistically significant and large. Because there were less than 10 studies, small-study effects were not assessed. With each study deleted from the model once, results remained statistically non-significant across all deletions. The associations ranged from approximately −0.01 to 0.02. Cumulative meta-analysis, ranked by the year the study started, demonstrated that results have been negative since the start of the first study in 1923 up to the sixth study that started in 1970 after which the cumulative results showed a positive association. Random-effects meta-regression revealed statistically significant evidence for an association between pooled ES (Fisher’s r to z score for the association between childhood obesity and adult TC) and follow-up age (β = −0.004, *p* = 0.04; *I* ^*2*^ reduced from 91% to 76%), length of follow-up (β = −0.004, *p* = 0.01; *I* ^*2*^ reduced from 91% to 67%), as well as the year of study onset (β = 0.003, *p* = 0.0045; *I* ^*2*^ reduced from 91% to 58%).

#### Total cholesterol (adjusted for adult adiposity)

When examining studies that adjusted for adult BMI, a negative and non-significant association was observed between childhood adiposity and adult TC (Table 2 and Fig. 8). The 95% PIs were non-significant as well. Heterogeneity was statistically significant and considered large. Because there were less than 10 studies, small-study effects were not assessed. With each study deleted from the model once, results remained statistically non-significant across all deletions except when one study by Barker et al., 2005 was deleted, resulting in a statistically significant and negative association [65]. The associations ranged from approximately −0.04 to −0.09. Cumulative meta-analysis, ranked by the year the studies started, demonstrated that results have been negative since examining the birth cohort of 1947. Random-effects meta-regression revealed statistically significant evidence for an association between pooled ES (Fisher’s r to z score for the association between childhood obesity and adult TC for studies that adjusted for adult BMI) and baseline age (β = −0.01, *p* < 0.0001; *I* ^*2*^ reduced from 86% to 0%) and sex (β = −0.32, *p* < 0.0001; *I* ^*2*^ reduced from 77% to 35%).

#### Low-density lipoprotein (unadjusted for adult adiposity)

Overall, there was a positive association between childhood adiposity and adult LDL (Table 2 and Fig. 9). However, the results were not statistically significant. The 95% PIs were non-significant as well. Heterogeneity was statistically significant and large. Because there were less than 10 studies, small-study effects were not assessed. With each study deleted from the model once, results remained statistically non-significant across all deletions. The associations ranged from approximately −0.004 to 0.049. Cumulative meta-analysis, ranked by the year the study started, demonstrated that results have been statistically non-significant since examining the birth cohort of 1947. The results were negative with the first three studies added and became positive after the fourth and fifth studies were included. Random-effects meta-regression revealed statistically significant evidence for an association between pooled ES (Fisher’s r to z score for the association between childhood obesity and adult LDL) and follow-up age (β = −0.005, *p* = 0.0001; *I* ^*2*^ reduced from 94% to 39%), length of follow-up (β = −0.004, *p* = 0.005; *I* ^*2*^ reduced from 94% to 59%), and the year of study onset (β = 0.004, *p* = 0.0007; *I* ^*2*^ reduced from 94% to 51%).

#### Low-density lipoprotein (adjusted for adult adiposity)

When examining the three studies that adjusted for adult BMI, a statistically significant and negative association was observed between childhood adiposity and adult LDL (Table 2 and Fig. 10). Heterogeneity was non-significant and 0% based on *I* ^*2*^. In addition, 95% PIs were non-significant. Because there were less than 10 studies, small-study effects were not assessed. With each study deleted from the model once, results remained statistically significant across all deletions. The associations ranged from approximately −0.07 to −0.09. Cumulative meta-analysis, ranked by the year the studies started, demonstrated that results have been statistically significant since the first study was conducted in 1947. Random-effects meta-regression revealed no statistically significant evidence for an association between pooled ES (Fisher’s r to z score for the association between childhood obesity and adult LDL for studies that adjusted for adult BMI) and sex. Because of insufficient data, none of the other variables were examined.

#### High-density lipoprotein (unadjusted for adult adiposity)

Overall, there was a statistically significant and negative association between childhood adiposity and adult HDL (Table 2 and Fig. 11). Heterogeneity was also statistically significant and large. However, 95% PIs were non-significant. Because there were less than 10 studies, small-study effects were not assessed. With each study deleted from the model once, results remained statistically significant across all deletions except when one study by Schmidt et al., 2011 was deleted [82]. Deleting this study resulted in a negative non-significant association. The associations ranged from approximately −0.04 to −0.07. Cumulative meta-analysis, ranked by the year the study started, demonstrated that results have been statistically significant since examining the birth cohort of 1985. Random-effects meta-regression revealed no statistically significant evidence for an association between pooled ES (Fisher’s r to z score for the association between childhood obesity and adult HDL) and all the covariates examined.

#### High-density lipoprotein (adjusted for adult adiposity)

When examining studies that adjusted for adult BMI, a positive but non-significant association was observed between childhood adiposity and adult HDL (Table 2 and Fig. 12). Heterogeneity was statistically significant and considered large. However, 95% PIs were non-significant. Because there were less than 10 studies, small-study effects were not assessed. With each study deleted from the model once, results remained statistically non-significant across all deletions. The associations ranged from approximately −0.01 to 0.08. Cumulative meta-analysis, ranked by the year the studies started, demonstrated that results have been statistically non-significant since the first study was conducted in 1947. However, it is important to note that these results are based on only four studies and the direction of association changed from positive to negative and then positive over time. Random-effects meta-regression revealed statistically significant evidence for an association between pooled ES (Fisher’s r to z score for the association between childhood obesity and adult HDL for studies that adjusted for adult BMI) and follow-up age (β = −0.007, *p* = 0.004; *I* ^*2*^ reduced from 94% to 74%), length of follow-up (β = −0.007, *p* = 0.004; *I* ^*2*^ reduced from 94% to 76%), sex (males vs. female, β = −0.13, *p* = 0.01; *I* ^*2*^ reduced from 66% to 59%), and risk of bias assessment for low risk (β = 0.0068, *p* = 0.0002; *I* ^*2*^ reduced from 94% to 56%).

#### Triglycerides (unadjusted for adult adiposity)

Overall, there was a statistically significant and positive association between childhood adiposity and adult TG (Table 2 and Fig. 13). Heterogeneity was also statistically significant and large. However, 95% PIs were non-significant. Because there were less than 10 studies, small-study effects were not assessed. With each study deleted from the model once, results remained statistically significant across all deletions except when Freedman et al., 2001 and Schmidt et al., 2011 were deleted [4, 82]. The associations ranged from approximately 0.07 to 0.11. Cumulative meta-analysis, ranked by the year the study started, demonstrated that results have been statistically significant since examining the birth cohort of 1985. Random-effects meta-regression revealed statistically significant evidence for an association between pooled ES (Fisher’s r to z score for the association between childhood obesity and adult TG) and follow-up age (β = −0.009, *p* < 0.0001; *I* ^*2*^ reduced from 83% to 5%), length of follow-up (β = −0.007, *p* = 0.0001; *I* ^*2*^ reduced from 83% to 50%), and risk of bias assessment for low risk (β = 0.007, *p* = 0.0122; *I* ^*2*^ reduced from 83% to 75%).

#### Triglycerides (adjusted for adult adiposity)

When examining studies that adjusted for adult BMI, a negative association was observed between childhood adiposity and adult TG (Table 2 and Fig. 14). However, the association was not statistically significant. The 95% PIs were non-significant as well. Heterogeneity was statistically significant and considered large. Because there were less than 10 studies, small-study effects were not assessed. With each study deleted from the model once, results remained statistically non-significant across all deletions. The associations ranged from approximately −0.05 to −0.13. Cumulative meta-analysis, ranked by the year the studies started, demonstrated that results have been statistically non-significant since examining the cohort of 1947. Random-effects meta-regression revealed statistically significant evidence for an association between pooled ES (Fisher’s r to z score for the association between childhood obesity and adult TG for studies that adjusted for adult BMI) and follow-up age (β = 0.006, *p* = 0.05; *I* ^*2*^ reduced from 95% to 92%), length of follow-up (β = 0.005, *p* = 0.02; *I* ^*2*^ reduced from 95% to 90%) and sex (male vs. females, β = 0.14, *p* = 0.002; *I* ^*2*^ reduced from 60% to 45%).

#### Non-high-density lipoprotein

Insufficient data were available to analyze non-HDL.

#### Sensitivity analysis

The results of our sensitivity analysis for those outcomes using only BMI as the exposure showed similar findings (Table 3). The findings suggest that childhood obesity is significantly and positively associated with adult SBP (Zr = 0.10; 95% CI: 0.06, 0.13), DBP (Zr = 0.11; 95% CI: 0.07, 0.14), and TG (Zr = 0.10; 95% CI: 0.02, 0.17), and significantly and inversely associated with adult HDL (Zr = −0.06; 95% CI: -0.11, −0.01).

## Discussion

### Overall findings

The purpose of this study was to conduct a systematic review and meta-analysis of studies that have examined the association between childhood obesity and adult CVD risk factors (SBP, DBP, TC, HDL, LDL, non-HDL, and TG). The overall unadjusted findings suggest that childhood obesity is significantly and positively associated with adult SBP, DBP and TG and significantly and negatively associated with adult HDL. This interpretation is supported by: (1) 95% CI for overall results that do not include the null, (2) consistency of overall results when each study was deleted from the model once (influence analysis), (3) significance of results over a long time period in which the included studies were conducted (cumulative meta-analysis), and (4) non-significant small study effects.

When examining studies that adjusted for adult obesity, the overall findings suggest that the association was significant and negative for SBP, DBP, and LDL while the associations between childhood obesity and adult HDL and TG became non-significant when adult BMI was accounted for. However, it is important to point out that less than one third of studies adjusted for adult adiposity measures [4, 65, 74, 76, 78, 85]. For the studies that adjusted for adult BMI, the associations became reversed, suggesting that the association between childhood adiposity and adult CVD risk factors is potentially mediated by adult adiposity. The correlation coefficient for childhood adiposity from childhood to adulthood ranged from 0.3 to 0.8 (mean = 0.6, SD = 0.1), demonstrating a medium to strong tracking of adiposity across the lifespan. This is also consistent with previous research suggesting that children who are obese have a 40%–80% chance of becoming overweight or obese adults [16–18].

Several factors need to be considered when examining the results of this study. First, we used random-effects models that incorporate heterogeneity into the analysis. However, based on the fixed-effect model, we observed a moderate to large amount of heterogeneity in the vast majority of outcomes assessed. While a random-effects model incorporates heterogeneity into the analysis, it does not explain the sources of heterogeneity. Second, the 95% PIs were not statistically significant as they overlapped the null (0) and thus, give us less confidence in the overall results of the study based on 95% PI’s [86, 87]. Third, many studies were considered to be at an increased risk of bias based on several items from the STROBE instrument (Fig. 2 and Additional file 6). However, while the study used the term ‘risk of bias’, the STROBE instrument provides more information about the quality of reporting versus the quality of the study. More specifically, nearly 70% of the studies were considered to be at a high risk of bias for the following elements: (1) describing any efforts to address potential sources of bias, (2) explaining how missing data were addressed, (3) explaining how loss to follow-up was addressed, (4) describing any sensitivity analyses, (5) providing reasons for non-participation at each stage, (6) considering the use of a flow diagram, (7) considering translating estimates of relative risk into absolute risk for a meaningful time period, (8) reporting and other analyses performed (analyses of subgroups and interactions, sensitivity analyses), and (9) not providing adequate information on participants characteristics. Loss to follow-up is one of the main sources of bias in longitudinal studies [88], with research suggesting that more than a 20% loss to follow-up as a potential threat to the internal validity of a study [89]. Only seven studies included information on loss to follow-up [4, 68, 69, 71, 72, 77, 82]. Lastly, some of the associations observed in our meta-regression analyses suggest that some factors may potentially effect the overall conclusions. These include different factors for different outcomes. The significant factors included: (1) baseline age for adult SBP and DBP, (2) follow up age for TC, LDL, and TG (3), length of follow-up for TC, LDL and TG, (4) the year of study onset for TC and LDL, and (5) risk of bias assessment for low risk for TG. For those studies that adjusted for adult BMI, factors included (1) baseline age for TC, (2) follow up age for SBP, DBP, HDL, and TG (3) length of follow-up for SBP, DBP, HDL, and TG (4) sex for TC, HDL, and TG (5) the year of study onset for DBP, and (6) the risk of bias assessment for low risk for HDL.

More specifically, results from our meta-regression analyses revealed that the association between childhood obesity and adult SBP and DBP increases as the baseline age increases. For TC, HDL, and LDL the association decreases as the follow-up age and length of follow-up increases and for TC and LDL the association increases as the year of study onset increases. For TG, only the association increased as the percent of low risk of bias increased. For studies that adjusted for adult BMI, the association between childhood obesity and adult TC increased as baseline age increased. For SBP, DBP, and HDL the association decreased as the follow-up age and length of follow-up increased and for HDL the association increased as the year of study onset increased. The association was lower in males compared to females for TC and HDL, and higher in males compared to females for TGs. For HDL, only the association increased as the percent of low risk of bias increased. However, one unusual finding was the association between childhood obesity and adult TG (adjusted for adult BMI) that increased with the increase in the follow-up age/length of follow-up. We hypothesize that this odd finding may be due to the play of chance given all the tests that were conducted. However, the results of the meta-regression tests should be interpreted with caution since they are considered observational inquiries to generate hypotheses about potential sources of heterogeneity [40] i.e. to explore which factors, if any, best account for changes in outcomes. Thus, these would need to be tested and confirmed in original studies.

### Evaluation of results compared to previous systematic reviews

As previously discussed, four systematic reviews [13, 21–23] published on this topic from 2010 to 2012 provided evidence on the association between childhood obesity and adult CVD risk factors (BP and lipid profile). While one study conducted quantitative analyses, it was limited to a select four cohorts only and thus, was not considered a true systematic review with meta-analysis [24]. The systematic review by Lloyd and colleagues [21] found little evidence that childhood obesity is an independent risk factor for adult SBP and DBP. Their study concluded that the relationships observed were dependent on the tracking of BMI from childhood to adulthood. They found that the positive association between childhood BMI and adult blood pressure was attenuated or became negative when taking adult BMI into account. The results of our study are in congruence with these findings. A second systematic review by Lloyd and colleagues [13] also found little evidence that childhood obesity is an independent risk factor adult TC, LDL, HDL, and TG. They found that the association between childhood BMI and adult lipid levels was attenuated or inversed when taking into account adult BMI. The results of our study are also consistent with the findings of this systematic review. The systematic review by Reilly and colleagues [23] reported a significant and positive association between childhood adiposity and adult HT. However, this systematic review did not report if the studies included in the review adjusted for adult adiposity. Park and colleagues [22] also found a significant and positive association between childhood adiposity and adult HT in their systematic review. Two out of five studies that adjusted for adult BMI and which were described in this systematic review [22] found no association. However Park and colleagues [22] examined HT while we examined SBP and DBP. Park and colleagues also suggested that since adult BMI is on the causal pathway for the association between childhood obesity and adult disease, adjusting for adult BMI has methodological limitations. One of the main limitations suggested was that adjustment for variables on the casual pathway can lead to spurious associations (over-adjustment biases) that can draw estimates towards the null. They also provided information from a previous study which showed a true positive association between birth weight and adult BP that was diminished after adjusting for current adult weight status, something that could be reversed if the correlation between birth weight and current weight was increased [90]. As childhood adiposity and adult adiposity are strongly correlated, this can be a potential problem. However, this debate has been both criticized by other researchers as well as supported [91–93]. Some of the main differences of our study from these earlier systematic reviews include: (1) combining the ESs of the included studies using the meta-analytic approach, (2) using SBP and DBP instead of HT [22, 23], (3) performing meta-analyses by systematically finding eligible studies for multiple risk factors, (4) including additional studies published up to June, 2015, (5) utilizing numerous definitions for childhood adiposity (exposure), (6) excluding studies that examined change of exposure from childhood to adulthood [13, 21], (7) excluding special populations [13, 21], (8) excluding gestational hypertension [23] and, (9) excluding studies that used self-reported outcomes [23].

A quantitative analysis by Juonala et al., [24] used data from four cohorts: the Bogalusa Heart Study (BHS) the Muscatine Study (MS), the Childhood Determinants of Adult Health (CDAH) study, and the Cardiovascular Risk in Young Finns Study (YFS). The results from this pooled, random-effects analysis showed a significant association between childhood obesity in predicting the following adult CVD outcomes using risk ratios: HT = 2.1 (95% CI: 1.8, 2.5), LDL = 1.6 (95% CI: 1.3, 2.0), high risk HDL = 1.7 (95% CI: 1.5, 1.9), and TG = 1.8 (95% CI: 1.5, 2.2). The direction of effect for the association between childhood obesity and adult CVD risk factors in the current meta-analysis is consistent with the previous work by Juonala et al. [24]. They also found a statistically significant association with HT even after adjustment for adult obesity (relative risk, 1.5; 95% CI: 1.1, 2.1; *P* = 0.009) [24]. For dyslipidemias, the effect of childhood adiposity was reduced and became non-significant when adult obesity was taken into account. The results of our meta-analysis are consistent with the pooled results for dyslipidemias. However, this previous study was not a true systematic review with meta-analysis [24]. Some of the main differences in our study compared to this previous investigation include: (1) using a systematic approach to find studies published until June 2015 that have examined these selected associations, (2) using SBP and DBP instead of HT, (3) examining the association for TC, (4) finding a positive but non-significant association for LDL, (5) performing a meta-analysis for numerous risk factors (SBP, DBP, TC, HDL LDL and TG), (6) performing a meta-analysis that adjusted for BMI, and (7) utilization of numerous definitions for childhood adiposity (exposure).

### Implications for research

The result of the current systematic review with meta-analysis has several implications for reporting and conducting future longitudinal studies. First, based on the STROBE instrument, it is recommended that future longitudinal studies improve their reporting with respect to several potential sources of bias. These include: (1) describing any efforts to address potential sources of bias, (2) explaining how missing data were addressed, (3) explaining how loss to follow-up was addressed, (4) describing any sensitivity analyses conducted, (4) reporting the numbers of individuals at each stage of the study, (5) providing reasons for non-participation at each stage, (6) considering the use of a flow diagram, (7) describing the characteristics of study participants, (8) considering translating estimates of relative risk into absolute risk for a meaningful time period, and (9) reporting any other analyses conducted (subgroups, interactions, and sensitivity analyses). Because longitudinal studies have a criterion for initially selecting participants that choose to participate or not, have varied response rates, different numbers of participants at baseline and follow-up, as well as varied participation and response rates at follow-up time point(s), it is important to provide this information using a flow diagram. However, only one study used a flow diagram. Therefore, it is suggested that future longitudinal studies include a flow diagram to clearly demonstrate their study design, participation and response rates. Second, complete information on the population characteristics should be presented, usually in Table 1, of most articles. Unfortunately, more than 50% of the studies did not provide adequate information on the population characteristics. Third, as loss to follow-up is a potential threat to the internal validity of a study [89], this information should be provided. Unfortunately, only seven studies included information on loss to follow-up. Fourth, only one study reported on the association between childhood obesity and adult non-HDL. This is important since non-HDL has been shown to be better predictor than LDL for coronary artery disease and stroke [25, 26]. Therefore, it is suggested that future studies collect and report this information. Fifth, only one third of the studies adjusted for adult adiposity. Given the former, it would appear prudent to suggest that future studies collect this information and present both crude and adjusted associations. Sixth, some studies presented results with unstandardized regression coefficients only. Among those studies that only provided unstandardized regression coefficients, this study was able to calculate standardized regression coefficients using the standard deviations of the exposure and the outcome. However, there were some studies where the standard deviations were not provided. As a result, we were unable to use data from these studies for our meta-analysis. Given this finding, it would appear prudent to suggest that future studies provide information for both standardized and unstandardized regression coefficients. Seventh, while the majority of studies included information on both males and females [66, 69, 72–75, 78, 79, 81, 84, 85], two were limited to men only [64, 71] while 9 combined data for both men and women [4, 65, 67, 68, 76–78, 82, 83]. Given biological differences between men and women, it would appear plausible to suggest that future studies include separate as well as combined results for both men and women. Eighth, although a wide range of confounders were accounted for in adjusted models, only four studies mentioned physical activity and energy intake during childhood and adulthood [69, 76, 81, 82] and only one study provided information on the pubertal status of children [72]. From our perspective, it is important to adjust for these variables as well as socio-demographic variables given previous research suggesting an association between pubertal timing and adult cardio-metabolic risk factors [94] as well as an association between energy intake, energy expenditure and adult CVD [95]. Ninth, most studies included in our study used BMI as a measure of adiposity in childhood [4, 65–69, 71, 72, 74–79, 81–85]. However, prior research has shown that BMI is not an ideal marker for adiposity [27, 28]. Therefore, it is suggested that future studies collect information on additional markers for adiposity, for example percent body fat, in addition to BMI. Additional use of an obesity index using age- and sex-specific thresholds might also provide more valid information regarding the effects of obesity on adult CVD. Tenth, the negative associations in the adjusted analysis for all outcomes, and a positive association for HDL provides the basis for future research to explore whether children at the lower end of BMI during childhood are at a higher risk for developing CVD risk factors compared to children at the higher end of the BMI spectrum during childhood and after adjusting for adult BMI. Lastly, while we may have been underpowered for some of our analyses, this should hopefully motivate researchers to include such information and/or analyses in their future studies. This is one of the very and often overlooked aspects of meta-analysis, that is, to provide direction for future research.

### Implications for practice

The result of the current systematic review with meta-analysis has relevant implications for practice. Overall, it appears that childhood obesity is positively associated with adult SBP, DBP, and TG and negatively associated with adult HDL. Although we did not evaluate the likelihood of a causal association using Hill’s criteria, several of these criteria (i.e. temporality, biological plausibility, coherence, experimental evidence, and analogy) suggest childhood obesity as a plausible risk factor for adult CVD risk factors [96]. Given the former, prevention of childhood obesity should remain a priority for public health interventions for preventing negative health outcomes during childhood as well as reducing the burden of adult obesity. Furthermore, this study provides important information to support the notion that obese children who become normal weight adults are probably not at any higher risk of CVD risk factor development if they become non-obese in adulthood. However, these findings need to be interpreted with caution given that only one third of the studies adjusted for adult BMI.

### Strengths of the current study

To the best of our knowledge, this is the most recent and complete study that has systematically appraised studies examining the associations included in this systematic review with meta-analysis. It is based on a greater number of studies (published up to June 2015) that included both crude associations as well as studies that adjusted for adult adiposity. This work also included any definition of adiposity and measure that was utilized for the exposure. This may be particularly relevant since it has been suggested that BMI is not an ideal marker for adiposity [27, 28]. From our perspective, including other definitions or classifications of adiposity helped us in identifying other potentially eligible studies that have looked at this association.

Although we performed the main meta-analysis using studies that utilized varied childhood adiposity measures, the results of our sensitivity analysis using only BMI as the exposure showed similar findings (Table 3). In addition, we also used SBP and DBP instead of HT to examine for independent associations between childhood obesity and components of HT (i.e. SBP and DBP). Lastly, we performed meta-regression analysis on covariates that may potentially impact this association and to inform future research on these factors.

### Potential limitations of the current study

The results of the current meta-analysis should be viewed with respect to the following potential limitations: (1) only one third of the included studies adjusted for adult BMI, (2) some of the pre-planned analyses to identify sources of heterogeneity were not performed due to lack of data, (3) the sample sizes for some of the analyses may have been underpowered to find a true effect, suggesting that future original studies may want to include such information, (4) due to the small sample sizes for some analyses, small-study effects (e.g. potential publication bias) were not conducted, (5) meta-analysis inherits the limitations of the original studies included, (6) a lack of empirical evidence, including assessment tools, for assessing study quality, especially given the difficulty in differentiating between quality of reporting and quality in the conduct of a study [32, 47–51] and (7) the inclusion of cohorts that ranged from 1923 to 1989 and the subsequent inability to assess if the relationship between childhood adiposity and adult cardiovascular risk might have changed over time as a result of changes in the treatment, management, and early identification of CVD risk over time. In addition, to retrieve information on missing data, we contacted the corresponding authors of the original studies via email. While 30% of the corresponding authors replied, no author provided any additional information. Furthermore, we excluded studies that were not published in the English language, and thus, may have introduced language bias. However, we do not believe that this was a major problem since previous research has shown that meta-analyses that restrict studies by language overestimate the effect of the outcomes by only 2% [41]. Also, like any systematic review, literature search bias is a potential problem where some relevant literature is not identified during the search process. However, we performed an exhaustive search according to pre-defined criteria, examining nearly 5000 citations. Thus, we expect any such bias to be minimal. Lastly, given the large number of analyses conducted, one or more of the study findings may have been due to the play of chance. However, no adjustment for multiple tests were made given that we did not want to miss potentially important findings that could be tested in future original studies [97].

## Conclusion

The results of this study suggest that childhood obesity may be a risk factor for selected adult cardiovascular disease risk factors. However, a need exists for additional, well-designed studies that include both unadjusted and adjusted data before any definitive conclusions can be reached.

## Additional files

Additional file 1: Search strategy to find existing systematic reviews with or without meta-analysis in PubMed (MEDLINE). (DOCX 173 kb)

Additional file 2: This file provides the previous systematic reviews and meta-analysis examining the association between childhood obesity and selected adult CVD risk factors including AMSTAR assessment of the previous systematic reviews and meta-analysis. (DOC 131 kb)

Additional file 3: PRISMA checklist (DOC 69 kb)

Additional file 4: Search strategy for electronic database. This file provides the search strategy used for the (1) PubMed (MEDLINE), (2) Web of Science, and (3) Scopus. (DOCX 574 kb)

Additional file 5: Excluded studies, including reasons. This file provides a reference list of all excluded studies, including the reasons for exclusion. (XLSX 900 kb)

Additional file 6: STROBE Checklist (DOCX 37 kb)

Additional file 7: Influence meta-analysis, cumulative meta-analysis and funnel plots (DOCX 3535 kb)

Additional file 8: Meta-Regression results for the association between childhood adiposity and adult CVD risk factors (DOCX 47 kb)

## Abbreviations

AMSTAR: Assessment of Multiple Systematic Reviews
BF: body fat
BHS: Bogalusa Heart Study
BMI: body mass index
BP: blood pressure
CDAH: the Childhood Determinants of Adult Health study
CI: confidence intervals
CVD: cardiovascular disease
DBP: diastolic blood pressure
ES: effect sizes
HDL: high-density lipoprotein cholesterol
HT: hypertension
LDL: low-density lipoprotein cholesterol
MS: the Muscatine Study
non-HDL: non-high-density lipoprotein cholesterol
OR: odds ratio
PI: prediction interval
PRISMA: Preferred Reporting Items for Systematic reviews and Meta-Analyses
SBP: systolic blood pressure
ST: skinfold thickness
STROBE: Strengthening the Reporting of Observational Studies in Epidemiology
TC: total cholesterol
TG: triglycerides
WC: waist circumference
WHR: waist to hip ratio
YFS: Cardiovascular Risk in Young Finns Study

## References

[1] Freedman DS, Mei Z, Srinivasan SR, Berenson GS, Dietz WH (2007). Cardiovascular risk factors and excess adiposity among overweight children and adolescents: the Bogalusa heart study. J Pediatr.

[2] Teixeira PJ, Sardinha LB, Going SB, Lohman TG (2001). Total and regional fat and serum cardiovascular disease risk factors in lean and obese children and adolescents. Obes Res.

[3] Celermajer DS, Ayer JG (2006). Childhood risk factors for adult cardiovascular disease and primary prevention in childhood. Heart.

[4] Freedman DS, Khan LK, Dietz WH, Srinivasan SR, Berenson GS (2001). Relationship of childhood obesity to coronary heart disease risk factors in adulthood: the Bogalusa heart study. Pediatrics.

[5] WHO: World health statistics 2009**.** Geneva: World Health Organization 2009.

[6] Mozaffarian D, Benjamin EJ, Go AS, Arnett DK, Blaha MJ, Cushman M, Das SR, de Ferranti S, Despres JP, Fullerton HJ (2016). Heart disease and stroke statistics-2016 update: a report from the American Heart Association. Circulation.

[7] Crowley DI, Khoury PR, Urbina EM, Ippisch HM, Kimball TR (2011). Cardiovascular impact of the pediatric obesity epidemic: higher left ventricular mass is related to higher body mass index. J Pediatr.

[8] Ford ES, Mokdad AH, Ajani UA (2004). Trends in risk factors for cardiovascular disease among children and adolescents in the United States. Pediatrics.

[9] Clinical Guidelines on the Identification, Evaluation, and Treatment of Overweight and Obesity in Adults--The Evidence Report. National Institutes of Health. Obesity research 1998, 6 Suppl 2:51S–209S. 9813653

[10] Freedman DS, Dietz WH, Tang R, Mensah GA, Bond MG, Urbina EM, Srinivasan S, Berenson GS (2004). The relation of obesity throughout life to carotid intima-media thickness in adulthood: the Bogalusa heart study. Int J Obes Relat Metab Disord.

[11] Baker JL, Olsen LW, Sorensen TI (2007). Childhood body-mass index and the risk of coronary heart disease in adulthood. N Engl J Med.

[12] Poirier P, Giles TD, Bray GA, Hong Y, Stern JS, Pi-Sunyer FX, Eckel RH, American Heart A (2006). Obesity Committee of the Council on nutrition PA, metabolism: obesity and cardiovascular disease: pathophysiology, evaluation, and effect of weight loss: an update of the 1997 American Heart Association scientific statement on obesity and heart disease from the obesity Committee of the Council on nutrition, physical activity, and metabolism. Circulation.

[13] Lloyd LJ, Langley-Evans SC, McMullen S (2012). Childhood obesity and risk of the adult metabolic syndrome: a systematic review. Int J Obes.

[14] Bridger T (2009). Childhood obesity and cardiovascular disease. Paediatr Child Health.

[15] Friedemann C, Heneghan C, Mahtani K, Thompson M, Perera R, Ward AM (2012). Cardiovascular disease risk in healthy children and its association with body mass index: systematic review and meta-analysis. BMJ.

[16] Fact sheets from the Surgeon General's Call to Action to Prevent and Decrease Overweight and Obesity. W V Med J. 2002;98(6):234–43. 12645272

[17] Deshmukh-Taskar P, Nicklas TA, Morales M, Yang SJ, Zakeri I, Berenson GS (2006). Tracking of overweight status from childhood to young adulthood: the Bogalusa heart study. Eur J Clin Nutr.

[18] Serdula MK, Ivery D, Coates RJ, Freedman DS, Williamson DF, Byers T (1993). Do obese children become obese adults? A review of the literature. Prev Med.

[19] Freedman DS, Khan LK, Serdula MK, Dietz WH, Srinivasan SR, Berenson GS (2004). Inter-relationships among childhood BMI, childhood height, and adult obesity: the Bogalusa heart study. Int J Obes Relat Metab Disord.

[20] Nadeau KJ, Maahs DM, Daniels SR, Eckel RH (2011). Childhood obesity and cardiovascular disease: links and prevention strategies. Nat Rev Cardiol.

[21] Lloyd LJ, Langley-Evans SC, McMullen S (2010). Childhood obesity and adult cardiovascular disease risk: a systematic review. Int J Obes.

[22] Park MH, Falconer C, Viner RM, Kinra S (2012). The impact of childhood obesity on morbidity and mortality in adulthood: a systematic review. Obes Rev.

[23] Reilly JJ, Kelly J (2011). Long-term impact of overweight and obesity in childhood and adolescence on morbidity and premature mortality in adulthood: systematic review. Int J Obes.

[24] Juonala M, Magnussen CG, Berenson GS, Venn A, Burns TL, Sabin MA, Srinivasan SR, Daniels SR, Davis PH, Chen W (2011). Childhood adiposity, adult adiposity, and cardiovascular risk factors. N Engl J Med.

[25] Virani SS (2011). Non-HDL cholesterol as a metric of good quality of care: opportunities and challenges. Tex Heart Inst J.

[26] Di Angelantonio E, Sarwar N, Perry P, Kaptoge S, Ray KK, Thompson A, Wood AM, Lewington S, Sattar N, Emerging Risk Factors C (2009). Major lipids, apolipoproteins, and risk of vascular disease. JAMA.

[27] Javed A, Jumean M, Murad MH, Okorodudu D, Kumar S, Somers VK, Sochor O, Lopez-Jimenez F. Diagnostic performance of body mass index to identify obesity as defined by body adiposity in children and adolescents: a systematic review and meta-analysis. Pediatr Obes. 2014; 10.1111/ijpo.242 24961794

[28] Brambilla P, Bedogni G, Heo M, Pietrobelli A (2013). Waist circumference-to-height ratio predicts adiposity better than body mass index in children and adolescents. Int J Obes.

[29] Shea BJ, Hamel C, Wells GA, Bouter LM, Kristjansson E, Grimshaw J, Henry DA, Boers M (2009). AMSTAR is a reliable and valid measurement tool to assess the methodological quality of systematic reviews. J Clin Epidemiol.

[30] Wright RW, Brand RA, Dunn W, Spindler KP (2007). How to write a systematic review. Clin Orthop Relat Res.

[31] Cook DJ, Mulrow CD, Haynes RB (1997). Systematic reviews: synthesis of best evidence for clinical decisions. Ann Intern Med.

[32] Higgins JPT, Green, S.: Cochrane Handbook for Systematic Reviews of Interventions Version 5.1.0., vol. Version 5.1.0.: The Cochrane Collaboration; 2011.; 2011.

[33] Reeves BC, Deeks, J. J., Higgins, J. P.T., Wells, G.A. : Chapter 13: Including non-randomized studies. In: Higgins JPT, Green S (editors), Cochrane Handbook for Systematic Reviews of Interventions. Version 5.0.1 The Cochrane Collaboration, 2008. Available from http://www.cochrane-handbook.org.; 2008.

[34] Moher D, Liberati A, Tetzlaff J, Altman DG (2009). Preferred reporting items for systematic reviews and meta-analyses: the PRISMA statement. BMJ.

[35] Panic N, Leoncini E, de Belvis G, Ricciardi W, Boccia S (2013). Evaluation of the endorsement of the preferred reporting items for systematic reviews and meta-analysis (PRISMA) statement on the quality of published systematic review and meta-analyses. PLoS One.

[36] (Available from http://www.crd.york.ac.uk/PROSPERO/display_record.asp?ID=CRD42015019763**).**

[37] Must A, Anderson SE (2006). Body mass index in children and adolescents: considerations for population-based applications. Int J Obes.

[38] Conn VS, Valentine JC, Cooper HM, Rantz MJ (2003). Grey literature in meta-analyses. Nurs Res.

[39] Rothstein HR, Sutton, A. J., Borenstein, M: Publication Bias in Meta-Analysis – Prevention, Assessment and Adjustments. 2005 John Wiley & Sons, Ltd; 2005.

[40] Littell JH, Corcoran J., Pillai, V.: Systematic reviews and meta-analysis, Oxford University Press, New York (2008), p. 202 ISBN: 978–0–19-532654-3; 2008.

[41] Moher D, Cook DJ, Eastwood S, Olkin I, Rennie D, Stroup DF (1999). Improving the quality of reports of meta-analyses of randomised controlled trials: the QUOROM statement. Quality of Reporting of Meta-analyses Lancet.

[42] Sampson M, McGowan J, Cogo E, Grimshaw J, Moher D, Lefebvre C (2009). An evidence-based practice guideline for the peer review of electronic search strategies. J Clin Epidemiol.

[43] EndNote [http://endnote.com/. In*.*

[44] Cohen J (1968). Weighted kappa: nominal scale agreement with provision for scaled disagreement or partial credit. Psychol Bull.

[45] Excel M (2011). Redmond.

[46] von Elm E, Altman DG, Egger M, Pocock SJ, Gotzsche PC, Vandenbroucke JP, Initiative S (2014). The strengthening the reporting of observational studies in epidemiology (STROBE) statement: guidelines for reporting observational studies. Int J Surg.

[47] Greenland S, O'Rourke K (2001). On the bias produced by quality scores in meta-analysis, and a hierarchical view of proposed solutions. Biostatistics.

[48] Ahn S (2016). Becker.

[49] Herbison P, Hay-Smith J, Gillespie WJ (2006). Adjustment of meta-analyses on the basis of quality scores should be abandoned. J Clin Epidemiol.

[50] Viswanathan M, Ansari MT, Berkman ND, Chang S, Hartling L, McPheeters M, Santaguida PL, Shamliyan T, Singh K, Tsertsvadze A (2008). Assessing the risk of bias of individual studies in systematic reviews of health care interventions.

[51] Deeks JJ, Dinnes J, D'Amico R, Sowden AJ, Sakarovitch C, Song F, Petticrew M, Altman DG: Evaluating non-randomised intervention studies. Health Technol Assess 2003, **7**(27):iii-x, 1–173. 10.3310/hta7270 14499048

[52] Borenstein M, Hedges, L. V., Higgins, J. P. T., Rothstein, H. R. : Introduction to Meta-Analysis. © 2009 John Wiley & Sons, Ltd. ISBN: 978–0–470-05724-7; 2009.

[53] Schellong K, Schulz S, Harder T, Plagemann A (2012). Birth weight and long-term overweight risk: systematic review and a meta-analysis including 643,902 persons from 66 studies and 26 countries globally. PLoS One.

[54] Peterson RA, Brown SP (2005). On the use of beta coefficients in meta-analysis. J Appl Psychol.

[55] DerSimonian R, Laird N (1986). Meta-analysis in clinical trials. Control Clin Trials.

[56] Higgins JP, Thompson SG (2002). Quantifying heterogeneity in a meta-analysis. Stat Med.

[57] Higgins JP, Thompson SG, Deeks JJ, Altman DG (2003). Measuring inconsistency in meta-analyses. BMJ.

[58] Egger M, Davey Smith G, Schneider M, Minder C (1997). Bias in meta-analysis detected by a simple, graphical test. BMJ.

[59] Sterne JA, Sutton AJ, Ioannidis JP, Terrin N, Jones DR, Lau J, Carpenter J, Rucker G, Harbord RM, Schmid CH (2011). Recommendations for examining and interpreting funnel plot asymmetry in meta-analyses of randomised controlled trials. BMJ.

[60] Higgins JP, Thompson SG, Spiegelhalter DJ (2009). A re-evaluation of random-effects meta-analysis. J Royal Stat Soc Series A.

[61] Song F, Khan KS, Dinnes J, Sutton AJ (2002). Asymmetric funnel plots and publication bias in meta-analyses of diagnostic accuracy. Int J Epidemiol.

[62] Higgins JP (2008). Commentary: heterogeneity in meta-analysis should be expected and appropriately quantified. Int J Epidemiol.

[63] Lau J, Schmid CH, Chalmers TC (1995). Cumulative meta-analysis of clinical trials builds evidence for exemplary medical care. J Clin Epidemiol.

[64] Abraham S, Collins G, Nordsieck M (1971). Relationship of childhood weight status to morbidity in adults. HSMHA health reports.

[65] Barker DJ, Osmond C, Forsen TJ, Kajantie E, Eriksson JG (2005). Trajectories of growth among children who have coronary events as adults. N Engl J Med.

[66] Berkey CS, Gardner J, Colditz GA (1998). Blood pressure in adolescence and early adulthood related to obesity and birth size. Obes Res.

[67] Eisenmann JC, Wickel EE, Welk GJ, Blair SN (2005). Relationship between adolescent fitness and fatness and cardiovascular disease risk factors in adulthood: the aerobics center longitudinal study (ACLS). Am Heart J.

[68] Graversen L, Sorensen TI, Petersen L, Sovio U, Kaakinen M, Sandbaek A, Laitinen J, Taanila A, Pouta A, Jarvelin MR (2014). Preschool weight and body mass index in relation to central obesity and metabolic syndrome in adulthood. PLoS One.

[69] Gustafsson PE, Persson M, Hammarstrom A (2011). Life course origins of the metabolic syndrome in middle-aged women and men: the role of socioeconomic status and metabolic risk factors in adolescence and early adulthood. Ann Epidemiol.

[70] Holland FJ, Stark O, Ades AE, Peckham CS (1993). Birth weight and body mass index in childhood, adolescence, and adulthood as predictors of blood pressure at age 36. J Epidemiol Community Health.

[71] Kanade A, Deshpande S, Patil K, Rao S (2011). Prevalence of high blood pressure among young rural adults in relation to height in childhood and adult body mass index. J Am Coll Nutr.

[72] Klumbiene J, Sileikiene L, Milasauskiene Z, Zaborskis A, Shatchkute A (2000). The relationship of childhood to adult blood pressure: longitudinal study of juvenile hypertension in Lithuania. J Hypertens.

[73] Kneisley J, Schork N, Julius S (1990). Predictors of blood pressure and hypertension in Tecumseh, Michigan. Clin Exp hypertension Part A, Theory and practice.

[74] Koziel S, Lipowicz A, Hulanicka B (2011). Childhood and adolescent body fat and its relationship with health outcome in 50 year old males and females: the Wroclaw growth study. Collegium antropologicum.

[75] Lauer RM, Clarke WR, Mahoney LT, Witt J (1993). Childhood predictors for high adult blood pressure. The Muscatine study. Pediatr Clin N Am.

[76] Li L, Law C, Power C (2007). Body mass index throughout the life-course and blood pressure in mid-adult life: a birth cohort study. J Hypertens.

[77] Liddle K, O'Callaghan M, Mamun A, Najman J, Williams G (2012). Comparison of body mass index and triceps skinfold at 5 years and young adult body mass index, waist circumference and blood pressure. J Paediatr Child Health.

[78] Lyngdoh T, Viswanathan B, van Wijngaarden E, Myers GJ, Bovet P (2013). Cross-sectional and longitudinal associations between body mass index and Cardiometabolic risk factors in adolescents in a country of the African region. Int J Endocrinol.

[79] Miura K, Nakagawa H, Tabata M, Morikawa Y, Nishijo M, Kagamimori S (2001). Birth weight, childhood growth, and cardiovascular disease risk factors in Japanese aged 20 years. Am J Epidemiol.

[80] Pinto Pereira SM, Power C (2013). Life course body mass index, birthweight and lipid levels in mid-adulthood: a nationwide birth cohort study. Eur Heart J.

[81] Porkka KV, Viikari JS, Taimela S, Dahl M, Akerblom HK (1994). Tracking and predictiveness of serum lipid and lipoprotein measurements in childhood: a 12-year follow-up. The cardiovascular risk in young Finns study. Am J Epidemiol.

[82] Schmidt MD, Dwyer T, Magnussen CG, Venn AJ (2011). Predictive associations between alternative measures of childhood adiposity and adult cardio-metabolic health. Int J Obes.

[83] Skidmore PM, Hardy RJ, Kuh DJ, Langenberg C, Wadsworth ME (2007). Life course body size and lipid levels at 53 years in a British birth cohort. J Epidemiol Community Health.

[84] Weitz CA, Friedlaender FY, Friedlaender JS (2014). Adult lipids associated with early life growth in traditional Melanesian societies undergoing rapid modernization: a longitudinal study of the mid-20th century. Am J Phys Anthropol.

[85] Wright CM, Parker L, Lamont D, Craft AW (2001). Implications of childhood obesity for adult health: findings from thousand families cohort study. BMJ.

[86] Guddat C, Grouven U, Bender R, Skipka G (2012). A note on the graphical presentation of prediction intervals in random-effects meta-analyses. Syt Rev.

[87] Riley RD, Higgins JP, Deeks JJ (2011). Interpretation of random effects meta-analyses. BMJ.

[88] Kristman V, Manno M, Cote P (2004). Loss to follow-up in cohort studies: how much is too much?. Eur J Epidemiol.

[89] Dettori JR (2011). Loss to follow-up. Evidence-based spine-care journal.

[90] Tu YK, West R, Ellison GT, Gilthorpe MS (2005). Why evidence for the fetal origins of adult disease might be a statistical artifact: the "reversal paradox" for the relation between birth weight and blood pressure in later life. Am J Epidemiol.

[91] Gillman MW (2005). Re: "why evidence for the fetal origins of adult disease might be a statistical artifact: the 'reversal paradox' for the relation between birth weight and blood pressure in later life". Am J Epidemiol.

[92] Cole TJ (2005). Re: "why evidence for the fetal origins of adult disease might be a statistical artifact: the 'reversal paradox' for the relation between birth weight and blood pressure in later life". Am J Epidemiol.

[93] Gillman MW (2002). Epidemiological challenges in studying the fetal origins of adult chronic disease. Int J Epidemiol.

[94] Prentice P, Viner RM (2013). Pubertal timing and adult obesity and cardiometabolic risk in women and men: a systematic review and meta-analysis. Int J Obes.

[95] Roberts CK, Barnard RJ (2005). Effects of exercise and diet on chronic disease. J Appl Physiol (1985).

[96] Hill AB (1965). The environment and disease: association or causation?. Proc R Soc Med.

[97] Rothman KJ (1990). No adjustments are needed for multiple comparisons. Epidemiology.

