# Supplementary material for: Childhood obesity and adult cardiovascular disease risk factors: a systematic review with meta-analysis
Source: BMC Public Health. 2017 Aug 29;17:683. doi: 10.1186/s12889-017-4691-z (PMC5575877; doi:10.1186/s12889-017-4691-z)

**Additional File 1- Search Strategy for Databases (February 2, 2015) to find existing systematic reviews and meta-analysis on the topic:**


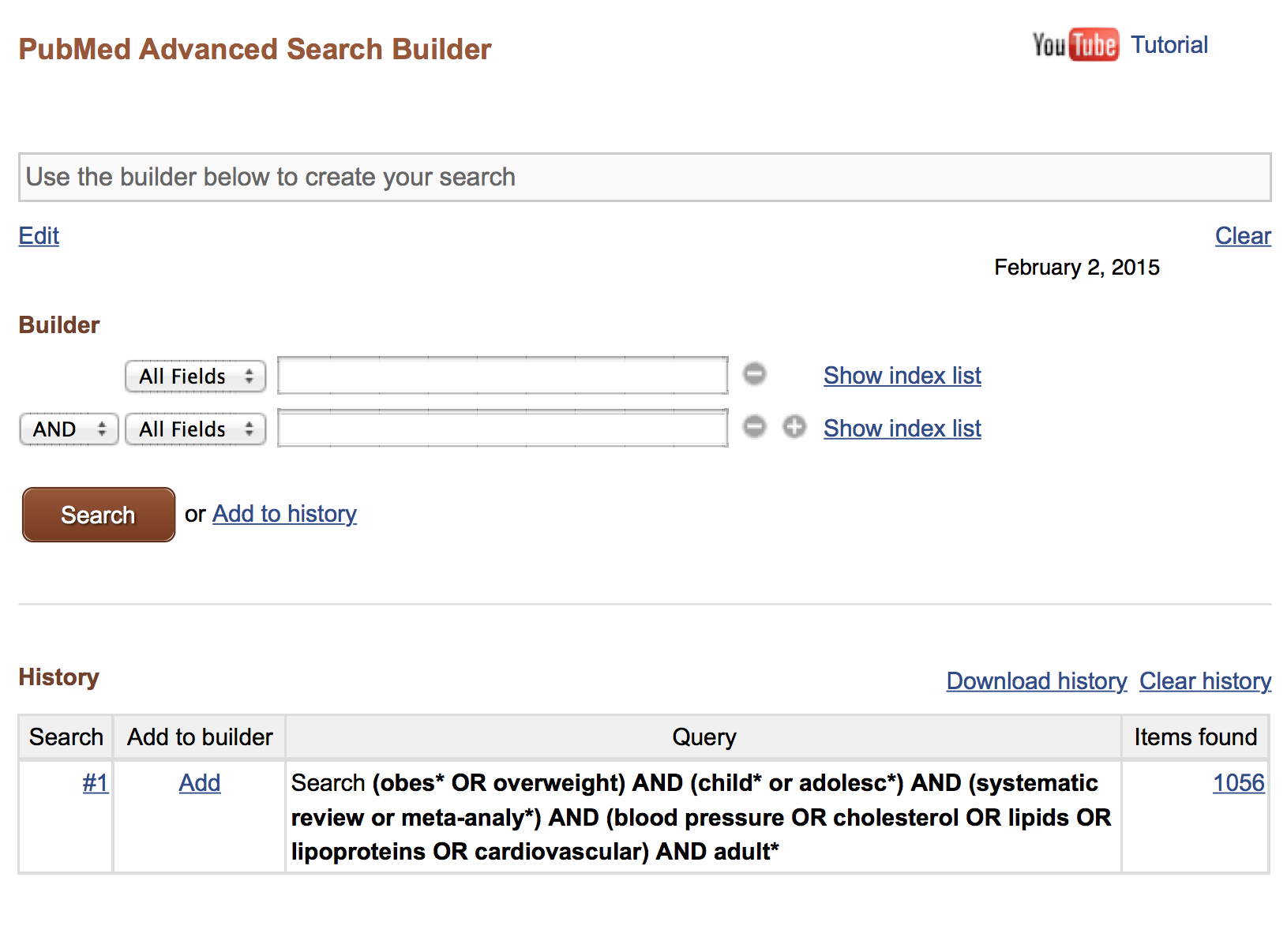

Supplement: Supplementary file 1 — Search strategy to find existing systematic reviews with or without meta-analysis in PubMed (MEDLINE). (DOCX 173 kb) [file 12889_2017_4691_MOESM1_ESM.docx]
