# Supplementary material for: Childhood obesity and adult cardiovascular disease risk factors: a systematic review with meta-analysis
Source: BMC Public Health. 2017 Aug 29;17:683. doi: 10.1186/s12889-017-4691-z (PMC5575877; doi:10.1186/s12889-017-4691-z)
Supplement: Supplementary file 2 — This file provides the previous systematic reviews and meta-analysis examining the association between childhood obesity and selected adult CVD risk factors including AMSTAR assessment of the previous systematic reviews and meta-analysis. (DOC 131 kb) [file 12889_2017_4691_MOESM2_ESM.doc]

**Additional File 2a:** Previous Systematic reviews (SR) and meta-analysis (MA) examining the association between childhood obesity and selected adult CVD risk factors

| Authors, year study published | Type of study design | No. of studies included and outcome of interest | Conclusion |
| --- | --- | --- | --- |
| Park et al., 2012 | SR | 5 studies for HT | Increase in BMI/overweight in childhood was associated with increased risk of HT. Two out of five studies that adjusted for adult BMI found no association. The review was unable to confirm the presence of long-term health effects of childhood obesity independent of its effects on adult BMI. This conclusion was not made for HT exclusively but the authors made a general statement. |
| Lloyd et al., 2012 | SR | 5 studies for lipids (TC, TG, LDL and HDL) | Little evidence that childhood obesity is an independent risk factor for adult blood lipid status. |
| Lloyd et al., 2010 | SR | 8 studies for blood pressure (SBP/DBP) | Little evidence that childhood obesity is an independent risk factor for CVD risk (SBP and DBP). The relationships observed are dependent on the tracking of BMI from childhood to adulthood. |
| Reilly et al., 2011 | SR | 4 studies for HT | Significant increase in adult HT with overweight and obesity during childhood and adolescence. The SR did not mention if the studies included controlled for adult weight status. |
| Juonala et al., 2011 | MA | Studies from 4-cohorts-BHS, MS, CDAH, and YFS | Childhood obesity was significantly associated with HT even after adjustment for adult obesity (relative risk, 1.5; 95% CI: 1.1, 2.1; P = 0.009). For dyslipidemias, the effect of childhood adiposity was reduced and became non-significant when adult obesity was taken into account. |

MA-Meta-analysis; SR-Systematic Review; CVD-Cardiovascular Disease; HT-hypertension; SBP-systolic blood pressure; DBP-diastolic blood pressure; TC-total cholesterol; TG-triglycerides; LDL-low-density lipoprotein cholesterol; HDL-high-density lipoprotein cholesterol; BHS-Bogalusa Heart Study; MS-Muscatine Study; CDAH-Childhood Determinants of Adult Health; YFS-Cardiovascular Risk in Young Finns Study

**Additional File 2b:** Overall score for each study (row) and item-by-item (column) results using the AMSTAR assessment instrument for previous SRs and MA

| **Authors** | **Year** | **1** | **2** | **3** | **4** | **5*** | **6** | **7** | **8** | **9** | **10** | **11** | **12** | **Total score Row (%)** |
| --- | --- | --- | --- | --- | --- | --- | --- | --- | --- | --- | --- | --- | --- | --- |
| Park et al., 2012 | 2012 | SR | Yes | Yes | Yes | No | Yes | Yes | Yes | No | NA | Yes | Yes | 8/10=80% |
| Lloyd et al., 2012 | 2012 | SR | Yes | Yes | Yes | No | No | Yes | Yes | Yes | NA | No | Yes | 7/10=70% |
| Lloyd et al., 2010 | 2010 | SR | Yes | Yes | Yes | No | No | Yes | Yes | Yes | NA | No | No | 6/10=60% |
| Reilly et al., 2011 | 2011 | SR | Yes | No | Yes | No | No | Yes | No | No | NA | No | Yes | 4/10=40% |
| Juonala et al., 2011 | 2011 | MA | Yes | No | NA | NA | NA | Yes | No | Yes | Yes | NA | Yes | 5/7=71% |
| Total score column (%) |  |  | 5/5=  100% | 3/5=  60% | 4/4=  100% | 0/4=  0% | 1/4=  25% | 5/5=  100% | 3/5=  60% | 3/5=  60% | 1/1=  100% | 1/4=  25% | 4/5=  80% |  |

Note: NA is “Not Applicable” is chosen when item is not relevant. Scores are adjusted for NA responses.

AMSTAR instrument:

1-Type of study design

2-Was a priori design provided?

3-Was there duplicate study selection and data extraction?

4-Was a comprehensive literature search performed?

5-Was the status of publication (i.e. grey literature) used as an inclusion criterion? *

6-Was a list of studies (included and excluded) provided?

7-Were the characteristics of the included studies provided?

8-Was the scientific quality of the included studies assessed and documented?

9-Was the scientific quality of the included studies used appropriately in formulating conclusions?

10-Were the methods used to combine the findings of studies appropriate?

11-Was the likelihood of publication bias assessed?

12-Was the conflict of interest included?

*This question was modified and is asking if the authors included grey literature as their inclusion criteria. ‘Yes’ means they did and ‘No’ means they did not.

References:

1. Park MH, Falconer C, Viner RM, Kinra S: **The impact of childhood obesity on morbidity and mortality in adulthood: a systematic review**. *Obes Rev* 2012, **13**(11):985-1000.

2. Israeli E, Korzets Z, Tekes-Manova D, Tirosh A, Schochat T, Bernheim J, Golan E: **Blood-pressure categories in adolescence predict development of hypertension in accordance with the European guidelines**. *Am J Hypertens* 2007, **20**(6):705-709.

3. Barker DJ, Eriksson JG, Forsen T, Osmond C: **Fetal origins of adult disease: strength of effects and biological basis**. *International journal of epidemiology* 2002, **31**(6):1235-1239.

4. Barker DJ, Forsen T, Eriksson JG, Osmond C: **Growth and living conditions in childhood and hypertension in adult life: a longitudinal study**. *Journal of hypertension* 2002, **20**(10):1951-1956.

5. Field AE, Cook NR, Gillman MW: **Weight status in childhood as a predictor of becoming overweight or hypertensive in early adulthood**. *Obesity research* 2005, **13**(1):163-169.

6. Li L, Law C, Power C: **Body mass index throughout the life-course and blood pressure in mid-adult life: a birth cohort study**. *Journal of hypertension* 2007, **25**(6):1215-1223.

7. Lloyd LJ, Langley-Evans SC, McMullen S: **Childhood obesity and risk of the adult metabolic syndrome: a systematic review**. *International journal of obesity* 2012, **36**(1):1-11.

8. Salonen MK, Kajantie E, Osmond C, Forsen T, Yliharsila H, Paile-Hyvarinen M, Barker DJ, Eriksson JG: **Role of childhood growth on the risk of metabolic syndrome in obese men and women**. *Diabetes Metab* 2009, **35**(2):94-100.

9. Freedman DS, Khan LK, Dietz WH, Srinivasan SR, Berenson GS: **Relationship of childhood obesity to coronary heart disease risk factors in adulthood: the Bogalusa Heart Study**. *Pediatrics* 2001, **108**(3):712-718.

10. Sinaiko AR, Donahue RP, Jacobs DR, Jr., Prineas RJ: **Relation of weight and rate of increase in weight during childhood and adolescence to body size, blood pressure, fasting insulin, and lipids in young adults. The Minneapolis Children's Blood Pressure Study**. *Circulation* 1999, **99**(11):1471-1476.

11. Wright CM, Parker L, Lamont D, Craft AW: **Implications of childhood obesity for adult health: findings from thousand families cohort study**. *Bmj* 2001, **323**(7324):1280-1284.

12. Lauer RM, Lee J, Clarke WR: **Factors affecting the relationship between childhood and adult cholesterol levels: the Muscatine Study**. *Pediatrics* 1988, **82**(3):309-318.

13. Lloyd LJ, Langley-Evans SC, McMullen S: **Childhood obesity and adult cardiovascular disease risk: a systematic review**. *International journal of obesity* 2010, **34**(1):18-28.

14. Hardy R, Wadsworth ME, Langenberg C, Kuh D: **Birthweight, childhood growth, and blood pressure at 43 years in a British birth cohort**. *International journal of epidemiology* 2004, **33**(1):121-129.

15. Lauer RM, Clarke WR: **Childhood risk factors for high adult blood pressure: the Muscatine Study**. *Pediatrics* 1989, **84**(4):633-641.

16. Burke V, Beilin LJ, Dunbar D, Kevan M: **Associations between blood pressure and overweight defined by new standards for body mass index in childhood**. *Prev Med* 2004, **38**(5):558-564.

17. Reilly JJ, Kelly J: **Long-term impact of overweight and obesity in childhood and adolescence on morbidity and premature mortality in adulthood: systematic review**. *International journal of obesity* 2011, **35**(7):891-898.

18. Ford CA, Nonnemaker JM, Wirth KE: **The influence of adolescent body mass index, physical activity, and tobacco use on blood pressure and cholesterol in young adulthood**. *J Adolesc Health* 2008, **43**(6):576-583.

19. Leeners B, Rath W, Kuse S, Irawan C, Neumaier-Wagner P: **The significance of under- or overweight during childhood as a risk factor for hypertensive diseases in pregnancy**. *Early Hum Dev* 2006, **82**(10):663-668.

20. Juonala M, Magnussen CG, Berenson GS, Venn A, Burns TL, Sabin MA, Srinivasan SR, Daniels SR, Davis PH, Chen W *et al*: **Childhood adiposity, adult adiposity, and cardiovascular risk factors**. *The New England journal of medicine* 2011, **365**(20):1876-1885.
