# Supplementary material for: Childhood obesity and adult cardiovascular disease risk factors: a systematic review with meta-analysis
Source: BMC Public Health. 2017 Aug 29;17:683. doi: 10.1186/s12889-017-4691-z (PMC5575877; doi:10.1186/s12889-017-4691-z)

**Additional File 4- Search Strategy for Databases (June 5, 2015)**

PubMed Database Search:


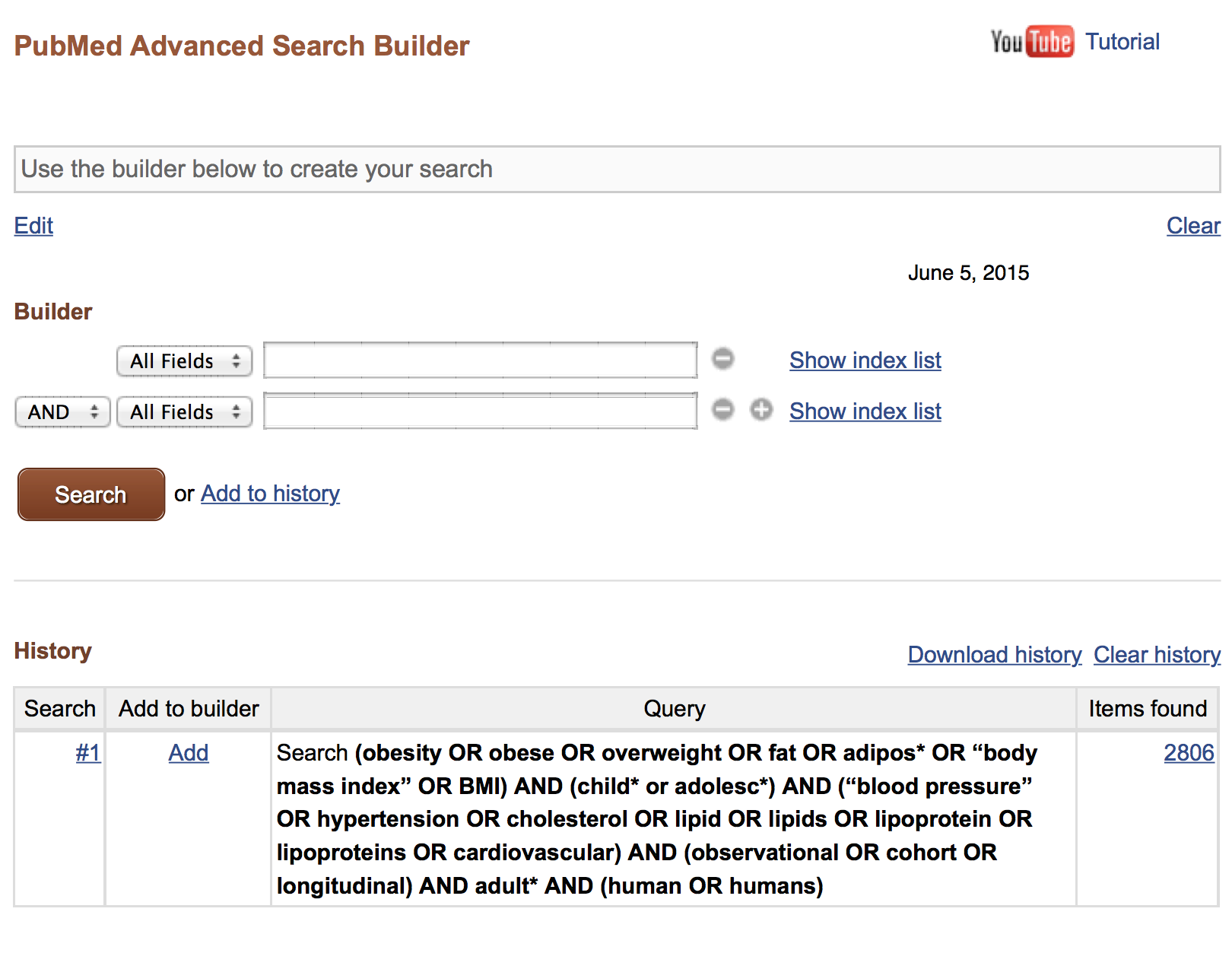


Web of Science Database Search


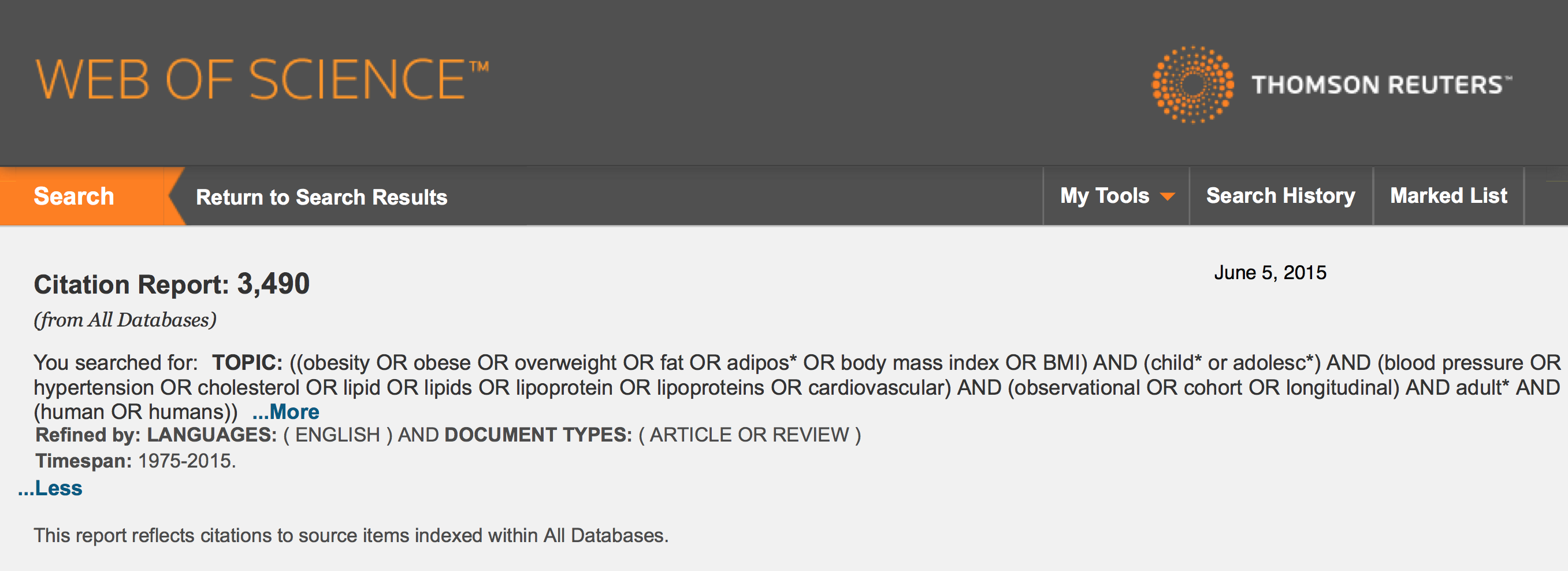


Scopus Database Search


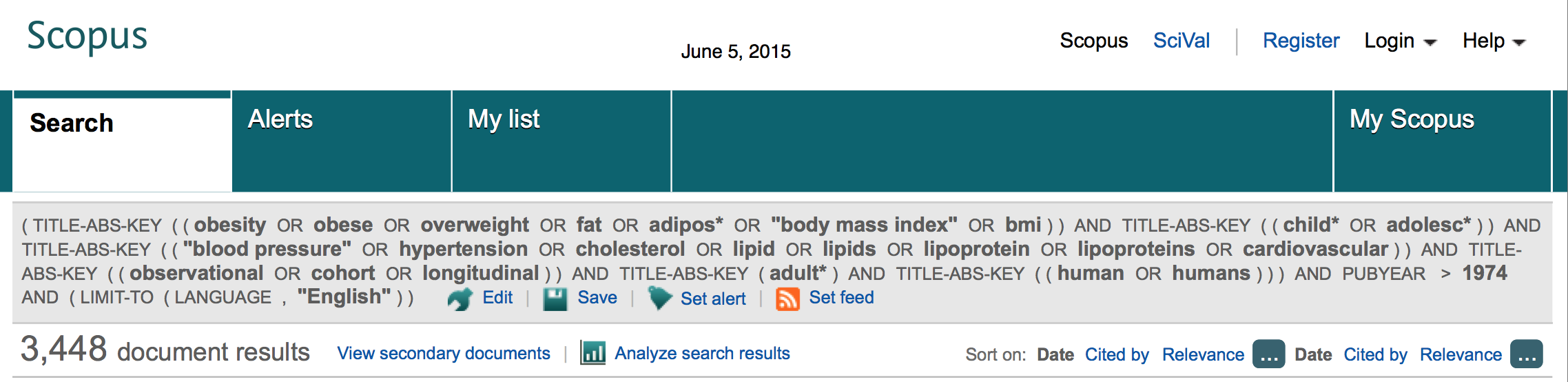

Supplement: Supplementary file 4 — Search strategy for electronic database. This file provides the search strategy used for the (1) PubMed (MEDLINE), (2) Web of Science, and (3) Scopus. (DOCX 574 kb) [file 12889_2017_4691_MOESM4_ESM.docx]
