# Supplementary material for: Childhood obesity and adult cardiovascular disease risk factors: a systematic review with meta-analysis
Source: BMC Public Health. 2017 Aug 29;17:683. doi: 10.1186/s12889-017-4691-z (PMC5575877; doi:10.1186/s12889-017-4691-z)
Supplement: Supplementary file 6 — STROBE Checklist (DOCX 37 kb) [file 12889_2017_4691_MOESM6_ESM.docx]

**Additional file 6: STROBE Checklist**

| **STROBE items** | **1 (a)** | **1 (b)** | **2** | **3** | **4** | **5** | **6 (a)** | **6 (b)** | **7** | **8** | **9** | **10** | **11** | **12 (a)** | **12 (b)** | **12 ('c)** | **12 (d)** |
| --- | --- | --- | --- | --- | --- | --- | --- | --- | --- | --- | --- | --- | --- | --- | --- | --- | --- |
| Abraham et al., 1971 | Low | Low | High | High | High | High | High | NA | High | High | High | High | High | Low | High | Low | High |
| Berkey et al., 1998 | High | High | High | High | High | High | Low | NA | High | High | Low | Low | Low | High | Low | Low | Low |
| Barker et al., 2005 | High | High | High | High | High | High | High | NA | High | High | Low | High | Low | High | High | Low | Low |
| Eisenmann JC et al., 2005 | High | High | High | High | High | High | High | NA | High | High | Low | High | High | High | High | Low | Low |
| Freedman DS et al., 2001 | High | High | High | High | High | High | High | NA | High | High | Low | High | High | High | High | Low | Low |
| Gustafsson et al., 2011 | High | High | High | High | High | High | High | NA | High | High | High | High | High | High | High | High | High |
| Graversen et al, 2014 | High | High | High | High | High | High | High | NA | High | High | Low | High | High | High | High | Low | Low |
| Holland et al., 1993 | High | High | High | High | High | High | High | NA | High | High | High | High | High | Low | Low | High | Low |
| Kanade et al., 2011 | High | High | High | High | High | High | High | NA | High | High | Low | High | High | Low | Low | Low | Low |
| Klumbiene et al., 2000 | High | High | High | High | High | High | High | NA | High | High | Low | High | High | High | High | Low | Low |
| Kneisley et al., 1990 | Low | High | Low | High | Low | Low | High | NA | Unclear | Low | Low | High | High | Low | Low | Low | Low |
| Koziel et al., 2011 | High | High | High | High | High | High | Low | NA | High | High | Low | Unclear | High | High | Low | Low | Low |
| Lauer et al., 1993 | Low | Low | High | High | High | High | High | NA | High | High | High | High | High | High | High | Low | Low |
| Li et al., 2007 | High | High | High | High | High | High | High | NA | High | High | Low | High | High | High | High | Low | Low |
| Liddle et al., 2012 | High | High | High | High | High | High | High | NA | High | High | High | High | High | Unclear | Low | Low | High |
| Lyngdoh et al., 2013 | High | High | High | High | High | High | High | NA | High | High | Low | High | High | High | High | High | Low |
| Miura et al., 2001 | High | High | High | High | High | High | High | NA | High | High | Low | High | NA | High | High | Low | Unclear |
| Pereira et al., 2013 | High | High | High | High | High | High | High | NA | High | High | High | High | High | High | High | High | Low |
| Porkka et al., 1994 | High | High | High | High | High | High | High | NA | High | Low | Low | High | Low | High | High | Low | Low |
| Schmidt et al., 2011 | High | High | High | High | High | High | High | NA | High | High | Low | High | High | High | High | Low | High |
| Skidmore et al., 2007 | High | High | High | High | High | High | High | NA | High | High | Low | Unclear | High | High | High | Low | Low |
| Weitz et al., 2014 | High | High | High | High | High | High | High | NA | High | High | Low | High | High | High | High | Low | Low |
| Wright et al., 2001 | High | High | High | High | High | High | High | NA | Low | Low | Low | High | High | High | High | Low | Low |
| **STROBE items** | **12 (e)** | **13 (a)** | **13 (b)** | **13 ('c)** | **14 (a)** | **14 (b)** | **14 ('c)** | **15** | **16 (a)** | **16 (b)** | **16 ('c)** | **17** | **18** | **19** | **20** | **21** | **22** |
| Abraham et al., 1971 | Low | High | Low | Low | Low | High | High | High | High | High | Low | Low | High | Low | High | Unclear | Low |
| Berkey et al. , 1998 | Low | High | Low | Low | High | High | High | High | High | High | NA | High | High | High | High | Low | High |
| Barker et al. , 2005 | Low | Low | Low | Low | High | Low | High | High | High | High | NA | High | High | Unclear | High | High | High |
| Eisenmann JC et al., 2005 | Low | High | Low | Low | High | Low | High | High | Low | High | NA | Low | High | High | High | High | High |
| Freedman DS et al., 2001 | Low | High | Unclear | Low | High | Low | High | High | High | High | NA | High | High | High | High | High | High |
| Gustafsson et al., 2011 | Low | High | High | Low | High | High | High | High | High | High | NA | High | High | High | High | High | Low |
| Graversen et al, 2014 | High | High | High | High | High | High | High | High | High | High | Low | High | High | High | High | High | High |
| Holland et al., 1993 | Low | Low | Low | Low | Low | Low | Low | Low | High | High | High | High | High | High | High | High | Low |
| Kanade et al., 2011 | Low | Low | Low | Low | High | High | Low | Low | Low | High | Low | High | High | Low | High | High | High |
| Klumbiene et al., 2000 | Low | Low | Low | Low | Low | High | High | High | High | High | NA | High | High | Low | High | Unclear | Low |
| Kneisley et al., 1990 | Low | Low | Low | Low | Low | Low | Low | Low | Low | High | NA | High | High | Low | High | High | High |
| Koziel et al., 2011 | Low | Low | Low | Low | Low | Low | Low | High | Low | NA | NA | Low | High | Low | High | High | High |
| Lauer et al., 1993 | High | Low | Low | Low | Low | Low | Low | High | High | High | Low | High | High | Low | High | High | High |
| Li et al., 2007 | High | High | Low | Low | High | High | Low | High | High | High | NA | High | High | High | High | High | High |
| Liddle et al., 2012 | Low | Low | Low | Low | High | Low | Low | High | High | High | Low | Low | High | High | High | High | High |
| Lyngdoh et al., 2013 | High | High | Low | Low | High | Low | Low | High | High | High | Low | High | High | High | High | High | High |
| Miura et al., 2001 | Low | High | Low | Low | Low | Low | High | High | High | NA | NA | High | High | High | High | High | High |
| Pereira et al., 2013 | High | High | Low | Low | Low | High | Low | High | High | High | NA | High | High | High | High | High | High |
| Porkka et al., 1994 | Low | High | Low | Low | Low | High | High | Low | Unclear | NA | NA | High | High | Low | High | High | High |
| Schmidt et al., 2011 | Low | High | Low | Low | Low | High | High | High | High | High | Low | High | High | High | High | High | High |
| Skidmore et al., 2007 | High | Low | High | Low | Low | High | High | High | High | NA | NA | High | High | High | High | High | High |
| Weitz et al., 2014 | Low | High | High | Low | Low | High | High | High | High | NA | NA | High | High | High | High | High | Low |
| Wright et al., 2001 | Low | Low | High | Low | Low | Low | Low | Low | High | NA | NA | High | High | High | High | High | High |

NA-Not Applicable

**STROBE Statement—checklist of items that should be included in reports of observational studies**

1 a. Indicate the study’s design with a commonly used term in the title or the abstract

1 b. Provide in the abstract an informative and balanced summary of what was done and what was found

2. Explain the scientific background and rationale for the investigation being reported

3. State specific objectives, including any pre-specified hypotheses

4. Present key elements of study design early in the paper

5. Describe the setting, locations, and relevant dates, including periods of recruitment, exposure, follow-up, and data collection

6 a. Give the eligibility criteria, and the sources and methods of selection of participants. Describe methods of follow-up

6 b. For matched studies, give matching criteria and number of exposed and unexposed-*Not Applicable for any study therefore this item is not shown in the figure below*

7. Clearly define all outcomes, exposures, predictors, potential confounders, and effect modifiers. Give diagnostic criteria, if applicable

8. For each variable of interest, give sources of data and details of methods of assessment. Describe comparability of assessment methods if there is more than one group

9. Describe any efforts to address potential sources of bias

10. Explain how the study size was arrived at.

11. Explain how quantitative variables were handled in the analyses. If applicable, describe which groupings were chosen and why

12 a. Describe all statistical methods, including those used to control for confounding

12 b. Describe any methods used to examine subgroups and interactions

12 c. Explain how missing data were addressed

12 d. Cohort study—If applicable, explain how loss to follow-up was addressed

12 e. Describe any sensitivity analyses

13 a. Report numbers of individuals at each stage of study

13 b. Give reasons for non-participation at each stage

13 c. Consider use of a flow diagram

14 a. Give characteristics of study participants

14 b. Indicate numbers of participants with missing data for each variable of interest

14 c. Summaries follow-up time

15. Report numbers of outcome events or summary measures over time

16 a. Give unadjusted estimates and, if applicable, confounder-adjusted estimates and their precision. Make clear which confounders were adjusted for and why they were included

16 b. Report category boundaries when continuous variables were categorized

16 c. If relevant, consider translating estimates of relative risk into absolute risk for a meaningful time period

17. Report other analyses done—eg analyses of subgroups and interactions, and sensitivity analyses

18. Summaries key results with reference to study objectives

19. Discuss limitations of the study, taking into account sources of potential bias or imprecision. Discuss both direction and magnitude of any potential bias

20. Give a cautious overall interpretation of results considering objectives, limitations, and multiplicity of analyses, results from similar studies, and other relevant evidence

21. Discuss the generalizability (external validity) of the study results

22. Give the source of funding and the role of the funders for the present study and, if applicable, for the original study on which the present article is based
