# Supplementary material for: Childhood obesity and adult cardiovascular disease risk factors: a systematic review with meta-analysis
Source: BMC Public Health. 2017 Aug 29;17:683. doi: 10.1186/s12889-017-4691-z (PMC5575877; doi:10.1186/s12889-017-4691-z)
Supplement: Supplementary file 8 — Meta-Regression results for the association between childhood adiposity and adult CVD risk factors (DOCX 47 kb) [file 12889_2017_4691_MOESM8_ESM.docx]

**Additional File 8**

**Meta-Regression results for the association between childhood adiposity and adult cardiovascular disease (CVD) risk factors**

Table: Systolic Blood Pressure (SBP) and Diastolic Blood Pressure (DBP)

| **CVD risk factors** | | | | | | | | | |
| --- | --- | --- | --- | --- | --- | --- | --- | --- | --- |
|  | **SBP** | | | | **DBP** | | | | |
| **Covariate** | **# of**  **Studies** | **Beta ± SE** | **CI (95%)** | **P-value** | **# of Studies** | | **Beta ± SE** | **CI (95%)** | **P-value** |
| Baseline Age | 16 | 0.0104 ± 0.004 | **0.0025, 0.0183*** | 0.0096 | 14 | | 0.0096 ± 0.0039 | **0.0019, 0.0173*** | 0.0145 |
| Follow-up Age | 16 | -0.0006 ± 0.002 | -0.0045, 0.0033 | 0.7559 | 14 | | -0.0011 ± 0.0016 | -0.0042, 0.0021 | 0.5116 |
| Length of Follow-up | 16 | -0.0024 ± 0.002 | -0.0063, 0.0014 | 0.216 | 14 | | -0.0028 ± 0.0017 | -0.0062, 0.0006 | 0.1076 |
| Year of Study Onset | 16 | -0.0009 ± 0.001 | -0.0029, 0.0012 | 0.4141 | 14 | | 0.0013 ± 0.0011 | -0.0009, 0.0035 | 0.2423 |
| Type of Analysis | 16 | 0.0218 ± 0.0421 | -0.0607, 0.1043 | 0.6046 | 14 | | 0.0176 ± 0.0393 | -0.0594, 0.0946 | 0.6535 |
| Country | 16 | 0.0741 ± 0.0386 | -0.0015, 0.1497 | 0.0548 | 14 | | 0.014 ± 0.0412 | -0.0668, 0.0948 | 0.7342 |
| Sex^a^ |  | 0.0303 ± 0.029 | -0.0265, 0.0871 | 0.2964 |  | | -0.0304 ± 0.0294 | -0.0879, 0.0272 | 0.3008 |
| Exposure^a^ |  | 0.0476 ± 0.0245 | -0.0005, 0.0956 | 0.0524 |  | | 0.0156 ± 0.0258 | -0.0351, 0.0662 | 0.5465 |
| Risk of Bias (Low-Risk) | 16 | 0.0016 ± 0.0014 | -0.0043, 0.0013 | 0.2643 | 14 | | 0.0008 ± 0.0018 | -0.0027, 0.0043 | 0.6701 |
| **CVD risk factors adjusted for adult BMI** | | | | | | | | | |
|  | **SBP** | | | | **DBP** | | | | |
| Baseline Age | 6 | 0.0055 ± 0.0084 | -0.011, 0.0221 | 0.5118 | 5 | 0.0072 ± 0.0249 | | -0.0416, 0.0559 | 0.7723 |
| Follow-up Age | 6 | -0.0046 ± 0.0015 | **-0.0075, -0.0017*** | 0.0017 | 5 | -0.006 ± 0.0014 | | **-0.0087, -0.0032*** | 0.0001 |
| Length of Follow-up | 6 | -0.0038 ± 0.0014 | **-0.0066, -0.001*** | 0.0078 | 5 | -0.0061 ± 0.0014 | | **-0.0089, -0.0034*** | 0.0001 |
| Year of Study Onset | 6 | 0.0029 ± 0.0017 | -0.0004, 0.0062 | 0.0885 | 5 | 0.0047 ± 0.0019 | | **0.001, 0.0083*** | 0.0122 |
| Sex^a^ |  | 0.0409 ± 0.0432 | -0.0438, 0.1256 | 0.3435 |  | 0.0165 ± 0.045 | | -0.0717, 0.1047 | 0.7141 |
| Risk of Bias (Low-Risk) | 6 | -0.0036 ± 0.0025 | -0.0014, 0.0086 | 0.1542 | 5 | 0.0032 ± 0.0033 | | -0.0032, 0.0096 | 0.3295 |

Type of Analysis: Correlation/Beta coefficient vs. Mean Difference/OR/RR

Country: USA vs. Others

Sex: Male vs. Female

Exposure: BMI vs. Other Measures of Adiposity

^a,^Factors assessed using sub-group as the study of analysis instead of study as the unit of analysis

For categorical variables, less than three results for any one category were used as the cut-off for analysis

*Statistically significant (non-overlapping 95% CI)

Table: Total cholesterol (TC) and Low-density lipoprotein cholesterol (LDL)

| **CVD risk factors** | | | | | | | | | | |
| --- | --- | --- | --- | --- | --- | --- | --- | --- | --- | --- |
|  | **TC** | | | | | **LDL** | | | | |
| **Covariate** | **# of**  **Studies** | **Beta ± SE** | **CI (95%)** | | **P-value** | **# of Studies** | | **Beta ± SE** | **CI (95%)** | **P-value** |
|  |  |  |  | |  |  | |  |  |  |
| Baseline Age | 8 | -0.0017 ± 0.0086 | -0.0186, 0.0153 | | 0.8477 | 5 | | 0.0108 ± 0.0159 | -0.0204, 0.042 | 0.4965 |
| Follow-up Age | 8 | -0.0035 ± 0.0017 | **-0.0068, -0.0002*** | | 0.0379 | 5 | | -0.0049 ± 0.0013 | **-0.0073, -0.0024*** | 0.0001 |
| Length of Follow-up | 8 | -0.0036 ± 0.0014 | **-0.0064, -0.0008*** | | 0.0124 | 5 | | -0.0041 ± 0.0015 | **-0.007, -0.0013*** | 0.0047 |
| Year of Study Onset | 8 | 0.003 ± 0.001 | **0.0009, 0.005*** | | 0.0045 | 5 | | 0.004 ± 0.0012 | **0.0017, 0.0063*** | 0.0007 |
| Type of Analysis |  |  |  | |  |  | |  |  |  |
| Country | 8 | 0.0599 ± 0.0465 | -0.0313, 0.1511 | | 0.1978 |  | |  |  |  |
| ^a^Sex |  | 0.0055 ± 0.0464 | -0.0853, 0.0964 | | 0.905 |  | | 0.0163 ± 0.0833 | -0.147, 0.1795 | 0.8453 |
| ^a^Exposure |  | -0.0584 ± 0.0408 | -0.1383, 0.0216 | | 0.1526 |  | |  |  |  |
| Risk of Bias (Low-Risk) | 8 | 0.0034 ± 0.0041 | -0.0047, 0.0114 | | 0.4155 | 5 | | 0.0056 ± 0.0048 | -0.0039, 0.0151 | 0.2471 |
| **CVD risk factors adjusted for adult BMI** | | | | | | | | | | |
|  | **TC** | | | | | **LDL** | | | | |
| Baseline Age | 4 | -0.0125 ± 0.0027 | **-0.0179, -0.0071*** | 0.0001 | |  |  | |  |  |
| Follow-up Age | 4 | 0.0023 ± 0.0028 | -0.0031, 0.0077 | 0.4002 | |  |  | |  |  |
| Length of Follow-up | 4 | 0.0025 ± 0.0019 | -0.0012, 0.0062 | 0.1903 | |  |  | |  |  |
| Year of Study Onset | 4 | -0.0029 ± 0.0016 | -0.0061, 0.0002 | 0.0661 | |  |  | |  |  |
| *Sex |  | -0.3151 ± 0.0444 | **-0.4022, -0.228*** | 0.0001 | |  | 0.0797 ± 0.0651 | | -0.0479, 0.2072 | 0.2208 |
| Risk of Bias (Low-Risk) | 4 | 0.0021 ± 0.0034 | -0.0046, 0.0088 | 0.5358 | |  |  | |  |  |

Type of Analysis: Correlation/Beta coefficient vs. Mean Difference/OR/RR

Country: USA vs. Others

Sex: Male vs. Female

Exposure: BMI vs. Other Measures of Adiposity

^a^Factors assessed using sub-group as the study of analysis instead of study as the unit of analysis

For categorical variables, less than three results for any one category were used as the cut-off for analysis

*Statistically significant (non-overlapping 95% CI)

Table: High-density lipoprotein cholesterol (HDL) and triglycerides (TG)

| **CVD risk factors** | | | | | | | | | |
| --- | --- | --- | --- | --- | --- | --- | --- | --- | --- |
|  | **HDL** | | | | **TG** | | | | |
| **Covariate** | **# of**  **Studies** | **Beta ± SE** | **CI (95%)** | **P-value** | **# of Studies** | | **Beta ± SE** | **CI (95%)** | **P-value** |
|  |  |  |  |  |  | |  |  |  |
| Baseline Age | 8 | -0.0072 ± 0.0079 | -0.0227, 0.0083 | 0.3622 | 8 | | -0.0041 ± 0.0095 | -0.0227, 0.0146 | 0.6699 |
| Follow-up Age | 8 | 0.0014 ± 0.0016 | -0.0017, 0.0045 | 0.3754 | 8 | | -0.0091 ± 0.0016 | **-0.0122, -0.006*** | 0.0001 |
| Length of Follow-up | 8 | 0.0013 ± 0.0014 | -0.0015, 0.0041 | 0.3507 | 8 | | -0.0073 ± 0.0019 | **-0.011, -0.0037*** | 0.0001 |
| Year of Study Onset | 8 | -0.0007 ± 0.0015 | -0.0035, 0.0022 | 0.6422 | 8 | | 0.004 ± 0.0023 | -0.0005, 0.0086 | 0.0827 |
| Type of Analysis |  |  |  |  |  | |  |  |  |
| Country |  |  |  |  |  | |  |  |  |
| ^a^Sex |  | 0.0144 ± 0.0517 | -0.0868, 0.1157 | 0.7798 |  | | 0.0141 ± 0.0964 | -0.1749, 0.2031 | 0.884 |
| ^a^Exposure |  | 0.0231 ± 0.0232 | -0.0224, 0.0685 | 0.3197 |  | | -0.0237 ± 0.0329 | -0.0882, 0.0408 | 0.4715 |
| Risk of Bias (Low-Risk) | 8 | 0.0005 ± 0.0028 | -0.005, 0.0061 | 0.8512 | 8 | | 0.007 ± 0.0028 | **0.0015, 0.0125*** | 0.0122 |
| **CVD risk factors adjusted for adult BMI** | | | | | | | | | |
|  | **HDL** | | | | **TG** | | | | |
| Baseline Age | 4 | 0.0104 ± 0.0438 | -0.0755, 0.0963 | 0.8123 | 5 | -0.0208 ± 0.0112 | | -0.0427, 0.0011 | 0.0622 |
| Follow-up Age | 4 | -0.0069 ± 0.0024 | **-0.0115, -0.0022*** | 0.0036 | 5 | 0.0055 ± 0.0028 | | **0, 0.0109*** | 0.0493 |
| Length of Follow-up | 4 | -0.0068 ± 0.0024 | **-0.0115, -0.0022*** | 0.0041 | 5 | 0.0049 ± 0.0021 | | **0.0008, 0.0091*** | 0.0205 |
| Year of Study Onset | 4 | 0.0052 ± 0.0032 | -0.0011, 0.0116 | 0.1068 | 5 | -0.0042 ± 0.0024 | | -0.0089, 0.0005 | 0.0824 |
| ^a^Sex |  | -0.1345 ± 0.0528 | **-0.2381, -0.031*** | 0.0109 |  | 0.144 ± 0.0456 | | **0.0546, 0.2334*** | 0.0016 |
| Risk of Bias (Low-Risk) | 4 | 0.0068 ± 0.0019 | **0.0032, 0.0105*** | 0.0002 | 5 | -0.0035 ± 0.0054 | | -0.0142, 0.0072 | 0.5176 |

Type of Analysis: Correlation/Beta coefficient vs. Mean Difference/OR/RR

Country: USA vs. Others

Sex: Male vs. Female

Exposure: BMI vs. Other Measures of Adiposity

^a^Factors assessed using sub-group as the study of analysis instead of study as the unit of analysis

For categorical variables, less than three results for any one category were used as the cut-off for analysis

*Statistically significant (non-overlapping 95% CI)
