# Supplementary material for: Childhood obesity and adult cardiovascular disease risk factors: a systematic review with meta-analysis
Source: BMC Public Health. 2017 Aug 29;17:683. doi: 10.1186/s12889-017-4691-z (PMC5575877; doi:10.1186/s12889-017-4691-z)
Supplement: Supplementary file 7 — Influence meta-analysis, cumulative meta-analysis and funnel plots (DOCX 3535 kb) [file 12889_2017_4691_MOESM7_ESM.docx]

**Additional File 7:**

**Influence meta-analysis, Cumulative meta-analysis and Funnel plots**

**Influence Analysis:**

**Figure 1a. Influence analysis for the association between childhood obesity and adult SBP**

Influence analysis for point estimate changes in SBP with each corresponding study deleted from the model once. The vertical lines represent in the middle of each straight line represents Fishers Z while the left and right extremes of the lines represent the corresponding 95% confidence intervals. The middle of the black diamond represents the overall mean difference while the left and right extremes of the diamond represent the corresponding 95% confidence intervals. Results are ordered from smallest to largest reductions. Combined measures represent those studies in which males and females were combined, different age cohorts from each study were combined, multiple readings from the same cohort were combined, or one study using more than one exposure definition was combined.

**Figure 1b. Influence analysis for the association between childhood obesity and adult SBP adjusted for adult BMI**

Influence analysis for point estimate changes in SBP with each corresponding study deleted from the model once. The vertical lines represent in the middle of each straight line represents Fishers Z while the left and right extremes of the lines represent the corresponding 95% confidence intervals. The middle of the black diamond represents the overall mean difference while the left and right extremes of the diamond represent the corresponding 95% confidence intervals. Results are ordered from smallest to largest reductions. Combined measures represent those studies in which males and females were combined, different age cohorts from each study were combined, multiple readings from the same cohort were combined, or one study using more than one exposure definition was combined.

**Figure 2a. Influence analysis for the association between childhood obesity and adult DBP**

Influence analysis for point estimate changes in DBP with each corresponding study deleted from the model once. The vertical lines represent in the middle of each straight line represents Fishers Z while the left and right extremes of the lines represent the corresponding 95% confidence intervals. The middle of the black diamond represents the overall mean difference while the left and right extremes of the diamond represent the corresponding 95% confidence intervals. Results are ordered from smallest to largest reductions. Combined measures represent those studies in which males and females were combined, different age cohorts from each study were combined, multiple readings from the same cohort were combined, or one study using more than one exposure definition was combined.

**Figure 2b. Influence analysis for the association between childhood obesity and adult DBP adjusted for adult BMI**

Influence analysis for point estimate changes in DBP with each corresponding study deleted from the model once. The vertical lines represent in the middle of each straight line represents Fishers Z while the left and right extremes of the lines represent the corresponding 95% confidence intervals. The middle of the black diamond represents the overall mean difference while the left and right extremes of the diamond represent the corresponding 95% confidence intervals. Results are ordered from smallest to largest reductions. Combined measures represent those studies in which males and females were combined, different age cohorts from each study were combined, multiple readings from the same cohort were combined, or one study using more than one exposure definition was combined.

**Figure 3a. Influence analysis for the association between childhood obesity and adult TC**

Influence analysis for point estimate changes in TC with each corresponding study deleted from the model once. The vertical lines represent in the middle of each straight line represents Fishers Z while the left and right extremes of the lines represent the corresponding 95% confidence intervals. The middle of the black diamond represents the overall mean difference while the left and right extremes of the diamond represent the corresponding 95% confidence intervals. Results are ordered from smallest to largest reductions. Combined measures represent those studies in which males and females were combined, different age cohorts from each study were combined, multiple readings from the same cohort were combined, or one study using more than one exposure definition was combined.

**Figure 3b. Influence analysis for the association between childhood obesity and adult TC adjusted for adult BMI**

Influence analysis for point estimate changes in TC with each corresponding study deleted from the model once. The vertical lines represent in the middle of each straight line represents Fishers Z while the left and right extremes of the lines represent the corresponding 95% confidence intervals. The middle of the black diamond represents the overall mean difference while the left and right extremes of the diamond represent the corresponding 95% confidence intervals. Results are ordered from smallest to largest reductions. Combined measures represent those studies in which males and females were combined, different age cohorts from each study were combined, multiple readings from the same cohort were combined, or one study using more than one exposure definition was combined.

**Figure 4a. Influence analysis for the association between childhood obesity and adult LDL**

Influence analysis for point estimate changes in LDL with each corresponding study deleted from the model once. The vertical lines represent in the middle of each straight line represents Fishers Z while the left and right extremes of the lines represent the corresponding 95% confidence intervals. The middle of the black diamond represents the overall mean difference while the left and right extremes of the diamond represent the corresponding 95% confidence intervals. Results are ordered from smallest to largest reductions. Combined measures represent those studies in which males and females were combined, different age cohorts from each study were combined, multiple readings from the same cohort were combined, or one study using more than one exposure definition was combined.

**Figure 4b. Influence analysis for the association between childhood obesity and adult LDL adjusted for adult BMI**

Influence analysis for point estimate changes in LDL with each corresponding study deleted from the model once. The vertical lines represent in the middle of each straight line represents Fishers Z while the left and right extremes of the lines represent the corresponding 95% confidence intervals. The middle of the black diamond represents the overall mean difference while the left and right extremes of the diamond represent the corresponding 95% confidence intervals. Results are ordered from smallest to largest reductions. Combined measures represent those studies in which males and females were combined, different age cohorts from each study were combined, multiple readings from the same cohort were combined, or one study using more than one exposure definition was combined.

**Figure 5a. Influence analysis for the association between childhood obesity and adult HDL**

Influence analysis for point estimate changes in HDL with each corresponding study deleted from the model once. The vertical lines represent in the middle of each straight line represents Fishers Z while the left and right extremes of the lines represent the corresponding 95% confidence intervals. The middle of the black diamond represents the overall mean difference while the left and right extremes of the diamond represent the corresponding 95% confidence intervals. Results are ordered from smallest to largest reductions. Combined measures represent those studies in which males and females were combined, different age cohorts from each study were combined, multiple readings from the same cohort were combined, or one study using more than one exposure definition was combined.

**Figure 5b. Influence analysis for the association between childhood obesity and adult HDL adjusted for adult BMI**

Influence analysis for point estimate changes in HDL with each corresponding study deleted from the model once. The vertical lines represent in the middle of each straight line represents Fishers Z while the left and right extremes of the lines represent the corresponding 95% confidence intervals. The middle of the black diamond represents the overall mean difference while the left and right extremes of the diamond represent the corresponding 95% confidence intervals. Results are ordered from smallest to largest reductions. Combined measures represent those studies in which males and females were combined, different age cohorts from each study were combined, multiple readings from the same cohort were combined, or one study using more than one exposure definition was combined.

**Figure 6a. Influence analysis for the association between childhood obesity and adult TG**

Influence analysis for point estimate changes in TG with each corresponding study deleted from the model once. The vertical lines represent in the middle of each straight line represents Fishers Z while the left and right extremes of the lines represent the corresponding 95% confidence intervals. The middle of the black diamond represents the overall mean difference while the left and right extremes of the diamond represent the corresponding 95% confidence intervals. Results are ordered from smallest to largest reductions. Combined measures represent those studies in which males and females were combined, different age cohorts from each study were combined, multiple readings from the same cohort were combined, or one study using more than one exposure definition was combined.

**Figure 6b. Influence analysis for the association between childhood obesity and adult TG adjusted for adult BMI**

Influence analysis for point estimate changes in TG with each corresponding study deleted from the model once. The vertical lines represent in the middle of each straight line represents Fishers Z while the left and right extremes of the lines represent the corresponding 95% confidence intervals. The middle of the black diamond represents the overall mean difference while the left and right extremes of the diamond represent the corresponding 95% confidence intervals. Results are ordered from smallest to largest reductions. Combined measures represent those studies in which males and females were combined, different age cohorts from each study were combined, multiple readings from the same cohort were combined, or one study using more than one exposure definition was combined.

**Cumulative Meta-Analysis:**

**Figure 1a. Cumulative meta-analysis for the association between childhood obesity and adult SBP**

Cumulative meta-analysis, ordered by the year the study started. The vertical lines between each straight line represent Fishers Z while the left and right extremes of the vertical lines represent the corresponding 95% confidence intervals. The results of each corresponding study are pooled with all studies preceding it. The middle of the black diamond represents the overall Fishers Z while the left and right extremes of the diamond represent the corresponding 95% confidence intervals. Combined measures represent those studies in which male and female were combined, different age cohorts from each study were combined, multiple readings from the same cohort were combined, or one study using more than one exposure definition was combined.

**Figure 1b. Cumulative meta-analysis for the association between childhood obesity and adult SBP adjusted for adult BMI**

Cumulative meta-analysis, ordered by the year the study started. The vertical lines between each straight line represent Fishers Z while the left and right extremes of the vertical lines represent the corresponding 95% confidence intervals. The results of each corresponding study are pooled with all studies preceding it. The middle of the black diamond represents the overall Fishers Z while the left and right extremes of the diamond represent the corresponding 95% confidence intervals. Combined measures represent those studies in which male and female were combined, different age cohorts from each study were combined, multiple readings from the same cohort were combined, or one study using more than one exposure definition was combined.

**Figure 2a. Cumulative meta-analysis for the association between childhood obesity and adult DBP**

Cumulative meta-analysis, ordered by the year the study started. The vertical lines between each straight line represent Fishers Z while the left and right extremes of the vertical lines represent the corresponding 95% confidence intervals. The results of each corresponding study are pooled with all studies preceding it. The middle of the black diamond represents the overall Fishers Z while the left and right extremes of the diamond represent the corresponding 95% confidence intervals. Combined measures represent those studies in which male and female were combined, different age cohorts from each study were combined, multiple readings from the same cohort were combined, or one study using more than one exposure definition was combined.

**Figure 2b. Cumulative meta-analysis for the association between childhood obesity and adult DBP adjusted for adult BMI**

Cumulative meta-analysis, ordered by the year the study started. The vertical lines between each straight line represent Fishers Z while the left and right extremes of the vertical lines represent the corresponding 95% confidence intervals. The results of each corresponding study are pooled with all studies preceding it. The middle of the black diamond represents the overall Fishers Z while the left and right extremes of the diamond represent the corresponding 95% confidence intervals. Combined measures represent those studies in which male and female were combined, different age cohorts from each study were combined, multiple readings from the same cohort were combined, or one study using more than one exposure definition was combined.

**Figure 3a. Cumulative meta-analysis for the association between childhood obesity and adult TC**

Cumulative meta-analysis, ordered by the year the study started. The vertical lines between each straight line represent Fishers Z while the left and right extremes of the vertical lines represent the corresponding 95% confidence intervals. The results of each corresponding study are pooled with all studies preceding it. The middle of the black diamond represents the overall Fishers Z while the left and right extremes of the diamond represent the corresponding 95% confidence intervals. Combined measures represent those studies in which male and female were combined, different age cohorts from each study were combined, multiple readings from the same cohort were combined, or one study using more than one exposure definition was combined.

**Figure 3b. Cumulative meta-analysis for the association between childhood obesity and adult TC adjusted for adult BMI**

Cumulative meta-analysis, ordered by the year the study started. The vertical lines between each straight line represent Fishers Z while the left and right extremes of the vertical lines represent the corresponding 95% confidence intervals. The results of each corresponding study are pooled with all studies preceding it. The middle of the black diamond represents the overall Fishers Z while the left and right extremes of the diamond represent the corresponding 95% confidence intervals. Combined measures represent those studies in which male and female were combined, different age cohorts from each study were combined, multiple readings from the same cohort were combined, or one study using more than one exposure definition was combined.

**Figure 4a. Cumulative meta-analysis for the association between childhood obesity and adult LDL**

Cumulative meta-analysis, ordered by the year the study started. The vertical lines between each straight line represent Fishers Z while the left and right extremes of the vertical lines represent the corresponding 95% confidence intervals. The results of each corresponding study are pooled with all studies preceding it. The middle of the black diamond represents the overall Fishers Z while the left and right extremes of the diamond represent the corresponding 95% confidence intervals. Combined measures represent those studies in which male and female were combined, different age cohorts from each study were combined, multiple readings from the same cohort were combined, or one study using more than one exposure definition was combined.

**Figure 4b. Cumulative meta-analysis for the association between childhood obesity and adult LDL adjusted for adult BMI**

Cumulative meta-analysis, ordered by the year the study started. The vertical lines between each straight line represent Fishers Z while the left and right extremes of the vertical lines represent the corresponding 95% confidence intervals. The results of each corresponding study are pooled with all studies preceding it. The middle of the black diamond represents the overall Fishers Z while the left and right extremes of the diamond represent the corresponding 95% confidence intervals. Combined measures represent those studies in which male and female were combined, different age cohorts from each study were combined, multiple readings from the same cohort were combined, or one study using more than one exposure definition was combined.

**Figure 5a. Cumulative meta-analysis for the association between childhood obesity and adult HDL**

Cumulative meta-analysis, ordered by the year the study started. The vertical lines between each straight line represent Fishers Z while the left and right extremes of the vertical lines represent the corresponding 95% confidence intervals. The results of each corresponding study are pooled with all studies preceding it. The middle of the black diamond represents the overall Fishers Z while the left and right extremes of the diamond represent the corresponding 95% confidence intervals. Combined measures represent those studies in which male and female were combined, different age cohorts from each study were combined, multiple readings from the same cohort were combined, or one study using more than one exposure definition was combined.

**Figure 5b. Cumulative meta-analysis for the association between childhood obesity and adult HDL adjusted for adult BMI**

Cumulative meta-analysis, ordered by the year the study started. The vertical lines between each straight line represent Fishers Z while the left and right extremes of the vertical lines represent the corresponding 95% confidence intervals. The results of each corresponding study are pooled with all studies preceding it. The middle of the black diamond represents the overall Fishers Z while the left and right extremes of the diamond represent the corresponding 95% confidence intervals. Combined measures represent those studies in which male and female were combined, different age cohorts from each study were combined, multiple readings from the same cohort were combined, or one study using more than one exposure definition was combined.

**Figure 6a. Cumulative meta-analysis for the association between childhood obesity and adult TG**

Cumulative meta-analysis, ordered by the year the study started. The vertical lines between each straight line represent Fishers Z while the left and right extremes of the vertical lines represent the corresponding 95% confidence intervals. The results of each corresponding study are pooled with all studies preceding it. The middle of the black diamond represents the overall Fishers Z while the left and right extremes of the diamond represent the corresponding 95% confidence intervals. Combined measures represent those studies in which male and female were combined, different age cohorts from each study were combined, multiple readings from the same cohort were combined, or one study using more than one exposure definition was combined.

**Figure 6b. Cumulative meta-analysis for the association between childhood obesity and adult TG**

Cumulative meta-analysis, ordered by the year the study started. The vertical lines between each straight line represent Fishers Z while the left and right extremes of the vertical lines represent the corresponding 95% confidence intervals. The results of each corresponding study are pooled with all studies preceding it. The middle of the black diamond represents the overall Fishers Z while the left and right extremes of the diamond represent the corresponding 95% confidence intervals. Combined measures represent those studies in which male and female were combined, different age cohorts from each study were combined, multiple readings from the same cohort were combined, or one study using more than one exposure definition was combined.

**Funnel plots:**

**Figure 1: Funnel plot for the association between childhood obesity and adult SBP**

**Figure 1: Funnel plot for the association between childhood obesity and adult DBP**
